# Epigenetic modifiers promote mitochondrial biogenesis and oxidative metabolism leading to enhanced differentiation of neuroprogenitor cells

**DOI:** 10.1038/s41419-018-0396-1

**Published:** 2018-03-02

**Authors:** Martine Uittenbogaard, Christine A. Brantner, Anne Chiaramello

**Affiliations:** 1 Department of Anatomy and Regenerative Biology, George Washington University School of Medicine and Health Sciences, Washington, DC USA; 2 GW Nanofabrication and Imaging Center, Office of the Vice President for Research, George Washington University, Washington, DC USA

## Abstract

During neural development, epigenetic modulation of chromatin acetylation is part of a dynamic, sequential and critical process to steer the fate of multipotent neural progenitors toward a specific lineage. Pan-HDAC inhibitors (HDCis) trigger neuronal differentiation by generating an “acetylation” signature and promoting the expression of neurogenic bHLH transcription factors. Our studies and others have revealed a link between neuronal differentiation and increase of mitochondrial mass. However, the neuronal regulation of mitochondrial biogenesis has remained largely unexplored. Here, we show that the HDACi, sodium butyrate (NaBt), promotes mitochondrial biogenesis via the NRF-1/Tfam axis in embryonic hippocampal progenitor cells and neuroprogenitor-like PC12-NeuroD6 cells, thereby enhancing their neuronal differentiation competency. Increased mitochondrial DNA replication by several pan-HDACis indicates a common mechanism by which they regulate mitochondrial biogenesis. NaBt also induces coordinates mitochondrial ultrastructural changes and enhanced OXPHOS metabolism, thereby increasing key mitochondrial bioenergetics parameters in neural progenitor cells. NaBt also endows the neuronal cells with increased mitochondrial spare capacity to confer resistance to oxidative stress associated with neuronal differentiation. We demonstrate that mitochondrial biogenesis is under HDAC-mediated epigenetic regulation, the timing of which is consistent with its integrative role during neuronal differentiation. Thus, our findings add a new facet to our mechanistic understanding of how pan-HDACis induce differentiation of neuronal progenitor cells. Our results reveal the concept that epigenetic modulation of the mitochondrial pool prior to neurotrophic signaling dictates the efficiency of initiation of neuronal differentiation during the transition from progenitor to differentiating neuronal cells. The histone acetyltransferase CREB-binding protein plays a key role in regulating the mitochondrial biomass. By ChIP-seq analysis, we show that NaBt confers an H3K27ac epigenetic signature in several interconnected nodes of nuclear genes vital for neuronal differentiation and mitochondrial reprogramming. Collectively, our study reports a novel developmental epigenetic layer that couples mitochondrial biogenesis to neuronal differentiation.

## Introduction

In the last decade, epigenetic modification of global chromatin landscape has emerged as a key mechanism regulating gene expression in a temporal and spatial manner during neurogenesis^1–3^. Notably, the neurogenic phase is associated with a unique histone acetylation signature in neural stem/progenitor cells that favors neuronal fate, lineage progression, and differentiation^4^. In contrast, low levels of acetylation confer astrocytic differentiation potential, while intermediate histone acetylation levels support oligodendrocytic fate^5,6^. Such acetylation homeostasis is determined by the interplay between two classes of antagonistic enzymes, histone acetyltransferases (HATs) and histone deacetylases (HDACs), which transfer or remove, respectively, an acetyl moiety of a lysine residue mapping in the N-terminal tail of nucleosomal histones^7^. Consequently, histone acetylation leads to relaxation of chromatin structure eliciting onset of gene transcription, while histone deacetylation induces a transcriptionally repressed chromatin. The HAT enzymes, CREB-binding protein (CBP) and p300, are essential for normal neuronal development illustrated by early embryonic lethality of CBP/p300 knockout mice and their link to neurodevelopmental disorders^2,8^. In neurons, the HAT/HDAC enzymes also target non-histone proteins, such as transcription factors and cytoskeletal proteins, thereby modulating gene expression and microtubule-based organelle transport^9,10^.

Pharmacological manipulation of HDAC activities using pan-HDAC inhibitors (HDACis), such as sodium butyrate (NaBt), trichostatin A (TSA), and valproic acid (VPA) induces neuronal differentiation of embryonic or adult neural progenitors at the expanse of glial differentiation^11,12^. Given that they ameliorate neuronal differentiation and survival in various experimental mouse models for neurodevelopmental disorders, they are potential therapeutic tools for central nervous system disorders^13,14^. However, our knowledge about their mechanism of action remains limited. They are known to promote neuronal differentiation by stimulating the expression of cell cycle inhibitors and neurogenic basic helix-loop-helix (bHLH) transcription factors, such as Ngn-1, MATH-1, and NeuroD^11,12^.

Given our previous findings of a direct link between the mitochondrial mass and NeuroD6 during the early stages of neuronal differentiation^15–17^, we asked whether NaBt could enhance neuronal differentiation by stimulating mitochondrial biogenesis and inducing a metabolic shift toward oxidative phosphorylation (OXPHOS). We used our engineered neuroprogenitor-like PC12-NeuroD6 cells (hereafter referred to as PC12-ND6) and E17.5 hippocampal neurons expressing high levels of NeuroD6^18^. Embryonic NeuroD6 expression is triggered at a time when neuronal progenitor cells undergo cell cycle withdrawal and initiate neuronal glutamatergic differentiation in the cortex and hippocampus^19^. NeuroD6 restricts the proliferation potential of committed neural progenitors during neurogenesis^19–21^.

In this study, we provide evidence for a novel developmental epigenetic layer coupling mitochondrial biogenesis to neuronal differentiation. NaBt induces mitochondrial biogenesis and enhances the oxidative metabolism in neural progenitor cells. NaBt adapts mitochondrial morphology to maximize mitochondrial respiratory activity generated by OXPHOS. Our results demonstrate that epigenetic modulation of the mitochondrial pool prior to neurotrophic signaling dictates the efficiency of initiation of neuronal differentiation during the transition from progenitor to differentiating neuronal cells. CBP modulates the mitochondrial biomass in neuronal precursor cells, confirming the NaBt-mediated regulation of mitochondrial mass. Finally, our genome-wide analysis of the epigenetic mark H3K27ac associated with active transcription shows that NaBt induces histone acetylation in several interconnected nodes of nuclear-encoded genes involved in neuronal differentiation and mitochondrial reprogramming.

## Results

### NaBt increases the mitochondrial biomass in neuronal progenitor-like PC12-ND6 cells and E17.5 hippocampal neurons

To test whether NaBt could modulate the mitochondrial mass in a neuronal context, we used our in vitro neuronal cellular paradigm, PC12-ND6 cells and E17.5 hippocampal neurons, both recapitulating the early stages of neuronal differentiation^22^. We established the optimal NaBt concentration for PC12-ND6 cells (5 mM) and E17.5 hippocampal neurons (2 mM) to induce maximal acetylation levels with minimal cellular toxicity, and showed that NaBt preferentially acetylated the lysine residues of the histone H3 at positions 9 and 27 (Fig. S1). We also confirmed that 2 mM of NaBt triggers differentiation of E17.5 hippocampal neurons (Fig. S2A, B), in keeping with VPA-mediated differentiation of adult neural stem cells^11,12^.

**Fig. 1:**
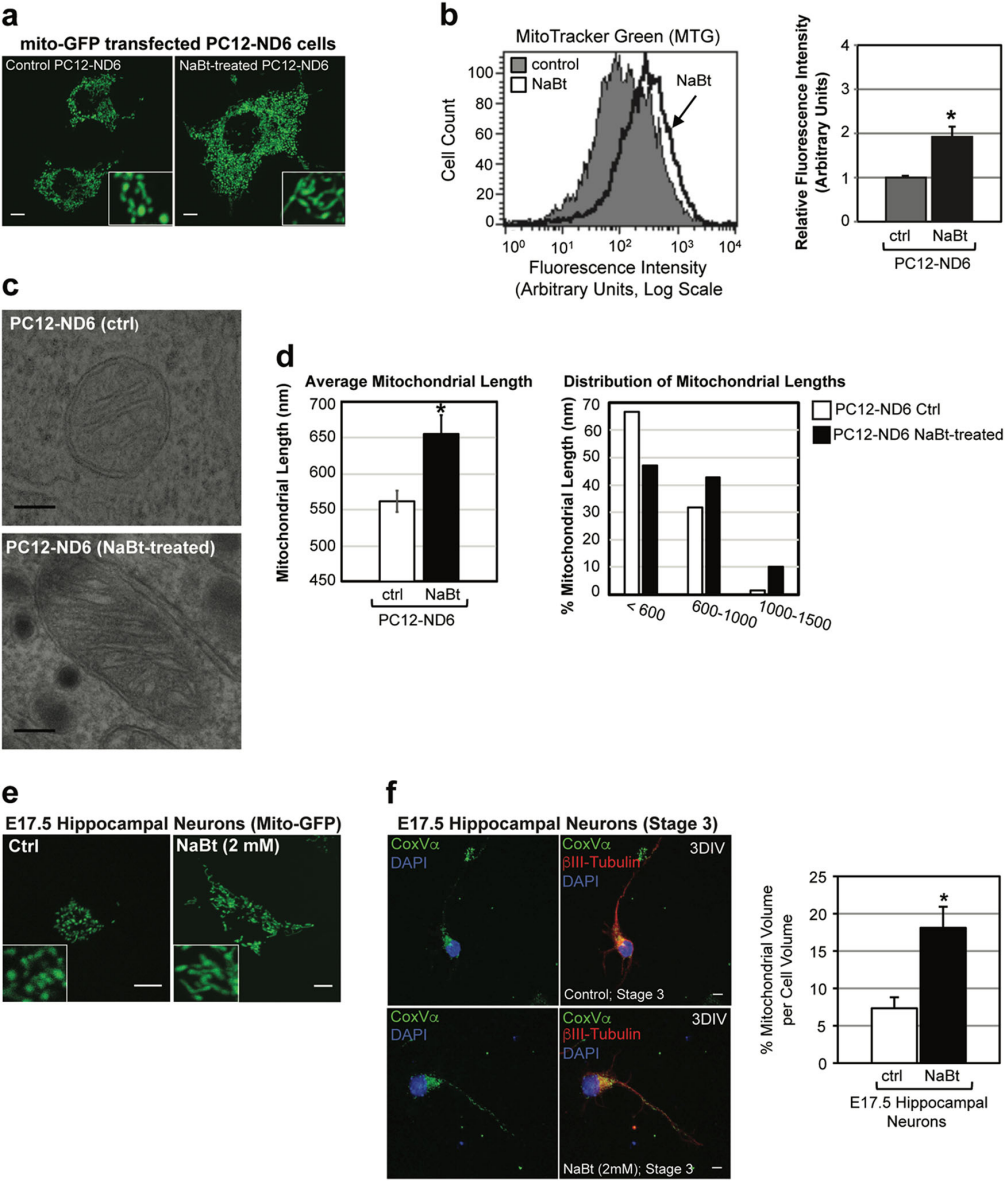
Sodium butyrate modulates the mitochondrial mass and mitochondrial microarchitecture in neuronal progenitor cells. **a** Representative live-cell confocal micrographs of PC12-ND6 cells transfected with the vector mito-GFP to visualize mitochondria in the absence or presence of NaBt (5 mM; 3 d). **b** Flow cytometry profiles of control and NaBt-treated PC12-ND6 cells stained with MitoTracker Green (MTG) and quantification of the fluorescence intensity of labeled control and NaBt-treated PC12-ND6 cells. **c** Representative electron micrographs of mitochondria in control (upper panel) and NaBt-treated (lower panel) PC12-ND6 cells (scale bar = 100 nm). **d** Quantification of average mitochondrial length (left panel) and distribution of mitochondrial lengths (right panel) in control and NaBt-treated PC12-ND6 cells. **e** Representative live-cell confocal analysis of the mitochondrial biomass of untreated and NaBt-treated E17.5 hippocampal neurons transfected with the vector mito-GFP at 3 DIV (scale bar = 5 µm). **f** Left panel showing confocal micrographs of control and NaBt-treated E17.5 hippocampal neurons at 3 DIV and stage 3 labeled with the pan-neuronal marker β-III tubulin (red), the mitochondrial marker COXVα (green), and the nuclear counterstain DAPI (blue). Scale bar = 5 µm. Right panel showing quantification of the percent of cell volume occupied by mitochondrial volume using stacked confocal images and Volocity software. All graphs are presented as mean ± S.D. *p ≤ 0.05 by *t*-test (*n* = 3)

We investigated whether NaBt could modulate the mitochondrial mass in PC12-ND6 cell and E17.5 neurons. Live-cell confocal microscopy revealed increased mitochondrial mass in NaBt-treated PC12-ND6 cells, which was quantified to a twofold increase by flow cytometry (Fig. 1a, b). NaBt induced an elongated mitochondrial morphology, which was confirmed by transmission electron microscopy revealing cristae remodeling with tightly stacked elongated cristae and reduced intercristae space (Fig. 1a, c, d). NaBt also increased the mitochondrial population in E17.5 hippocampal neurons by a 2.5 fold (Fig. 1f). While control E17.5 neurons have short rod-like mitochondria, NaBt-treated neurons harbor elongated mitochondria (Fig. 1e), an effect sustained up to 8 DIV (Fig. S2D). These results show that NaBt increases the mitochondrial population and triggers mitochondrial ultrastructural changes in progenitor neuronal cells.

### NaBt enhances the population of bioenergetic mitochondria and OXPHOS metabolism in PC12-ND6 cells

**Fig. 2:**
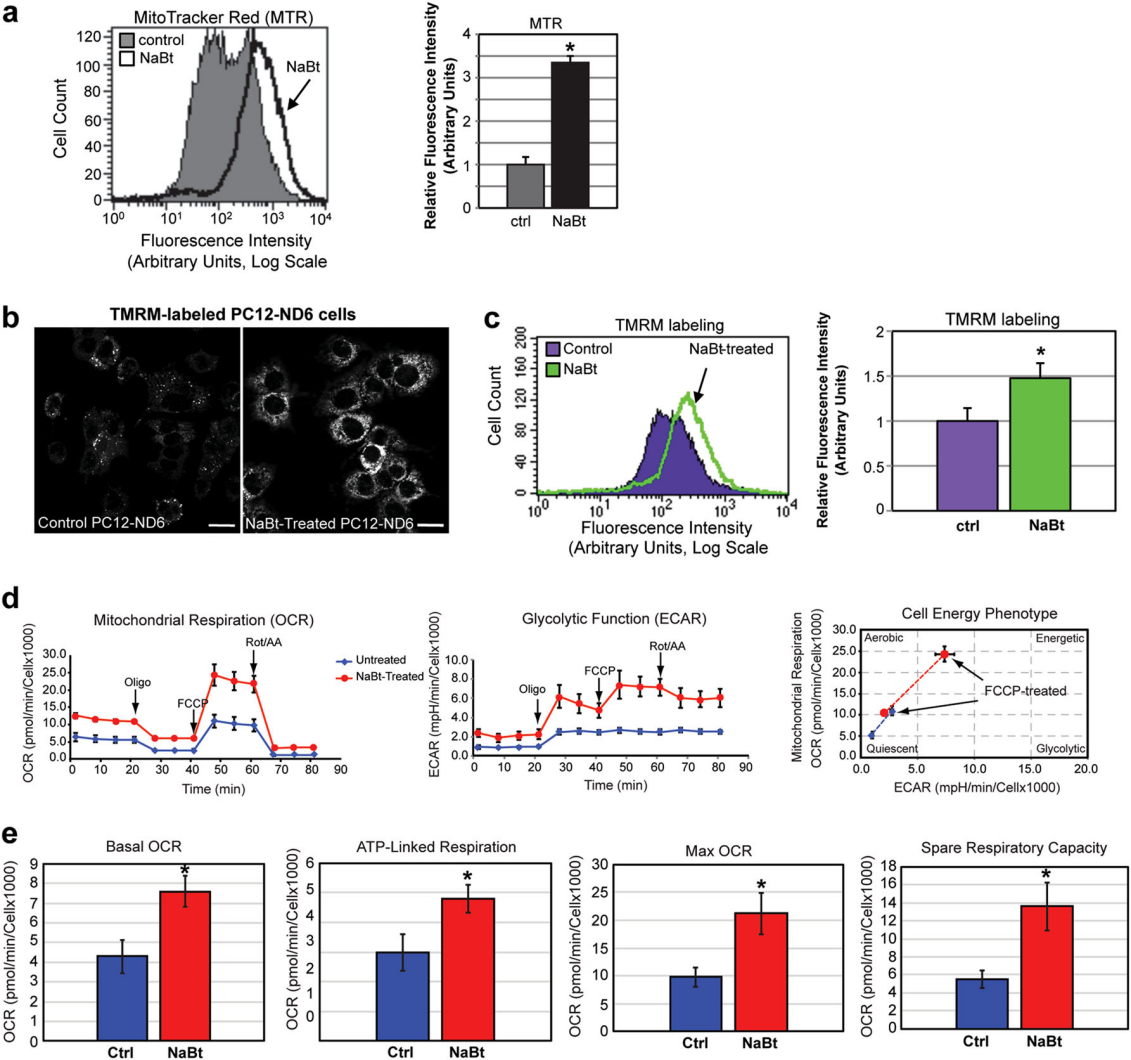
Sodium butyrate augments the mitochondrial bioenergetic capacity of PC12-ND6 cells. **a** Left panel showing flow cytometry profiles of control and NaBt-treated PC12-ND6 cells stained with MitoTracker Red (MTR) and right panel showing the quantification of the fluorescence intensity of labeled control and NaBt-treated PC12-ND6 cells stained with MTR. **b** Representative live-cell confocal micrograph of control and NaBt-treated PC12-ND6 cells staining with the TMRM dye to assess the mitochondrial membrane potential. Scale bar = 20 µm. **c** Left panel showing the flow cytometry analysis of the mitochondrial membrane potential in control and NaBt-treated PC12-ND6 cells stained with TMRM and the right panel showing the quantification of the TMRM fluorescence intensity. **d** The left panel shows a representative profile of oxygen consumption rate (OCR) from the mitochondrial stress test on duplicates from each cell type. The control and NaBt-treated PC12-ND6 cells are indicated in blue and red, respectively. The drugs added throughout the test are indicated on top of the graph, as described in Materials and methods. The middle panel shows a representative profile of extracellular acidification rate (ECAR) from the glycolytic function of the mitochondrial stress test. The left panel shows the overall cell phenotype profile from control (blue) and NaBt-treated PC12-ND6 cells (red). **e** Quantification of individual parameters for basal OCR, ATP-linked respiration, maximal OCR, and spare respiratory capacity. Results are from three independent experiments each using duplicate samples. All graphs are presented as mean ± S.D. *p ≤ 0.05 by* t*-test (*n* = 3)

We assessed whether NaBt could enhance mitochondrial bioenergetics in PC12-ND6 cells using the mitochondrial dyes MTR and MTG. While MTG solely stains mitochondria independently of the mitochondrial membrane potential (ΔΨ_m_), MTR labels energized mitochondria owing to its ΔΨ_m_-dependent retention in the mitochondrial matrix^15^. Flow cytometry analysis reveals a 3.5-fold and twofold increase in MTR and MTG signal, respectively (Fig. 2a), suggestive of increased ΔΨ_m_ by NaBt. This was corroborated with the dye tetramethylrhodamine methyl ester (TMRM), which accummulates in the mitochondrial matrix in a ΔΨ_m_-dependent fashion^17^. NaBt increases the TMRM signal by a 1.5 fold (Fig. 2b, c). We ruled out that NaBt increased ROS levels as a consequence of increased mitochondrial bioenergetics using the MitoSOX™ dye (Fig. S3).

These results led us to speculate that NaBt stimulates the OXPHOS metabolism. Using the Seahorse technology, we investigated the NaBt effect on the metabolic potential of PC12-ND6 cells by assessing mitochondrial OXPHOS and glycolysis. NaBt-treated cells exhibited increased mitochondrial respiration while glycolysis remained unchanged (Fig. 2d). We next measured key bioenergetic parameters of the OXPHOS pathway and found that the basal oxygen consumption rate (OCR) of NaBt-treated PC12-ND6 cells increased by 78% when compared to that of control PC12-ND6 cells (Fig. 2d, e). By exposing the cells to oligomycin A, an inhibitor of the ATP synthase, we detected a 56% increase in ATP-linked respiration in the presence of NaBt (Fig. 2d, e), in keeping with the increased ΔΨ_m_ (Fig. 2b, c). Using the uncoupler FCCP, we found that NaBt increased maximal and spare respiratory capacities by 115 and 145%, respectively, (Fig. 2d, e). NaBt-treated cells exhibited an FCCP-mediated increase in ECAR followed by a decreased ECAR triggered by antimycin A, consistent with respiratory acidification from increased tricarboxylic acid (TCA) cycle activities, resulting in greater CO2 levels (Fig. 2d). Thus, our bioenergetic results supports our confocal microscopy and flow cytometry analyses, revealing the ability of NaBt to generate a reserve of bioenergetically competent mitochondria with enhanced OXPHOS metabolism.

### NaBt induces mitochondrial biogenesis in PC12-ND6 cells

**Fig. 3:**
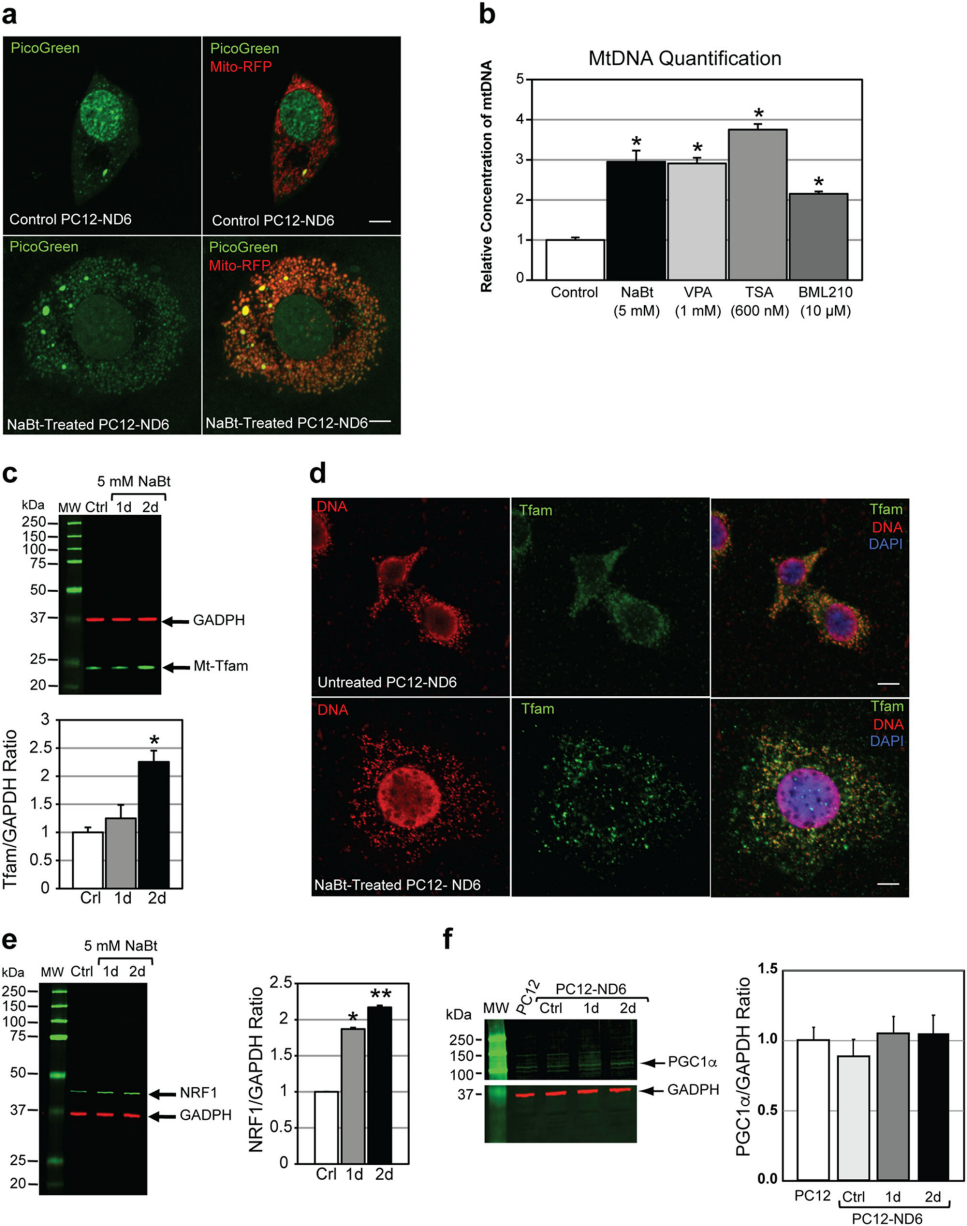
Sodium butyrate stimulates mitochondrial biogenesis in PC12-ND6 cells by increasing mt-DNA copy number and expression levels of key regulators of mt-DNA replication. **a** Representative live-cell confocal micrograph of PicoGreen staining of mt-nucleoids in control and NaBt-treated PC12-ND6 cells transfected with the mito-RFP vector. Scale bar = 5 µm. **b** Quantification of Mt-DNA copy number by qPCR from control and HDACi-treated PC12-ND6 cells and normalized by ΔΔ*C*(t). Mean ± SEM (*n* = 3; *p < 0.001). **c** Upper panel illustrating quantitative immunoblot analysis of TFAM expression in control and NaBt-treated PC12-ND6 cells. Lower panel showing the graphed TFAM/GAPDH ratio normalized to untreated PC12-ND6 cells. Mean ± SEM (*n* = 3; *p ≤ 0.05). **d** Representative confocal micrographs of control and NaBt-treated PC12-ND6 cells stained for mitochondrial-specific transcriptional factor TFAM (green), mt-DNA (red), and the nuclear counterstain DAPI (blue). Scale bar = 5 µm. **e** Left panel illustrating the quantitative immunoblot analysis of NRF-1 expression in control and NaBt-treated PC12-ND6 cells. Right panel showing the graphed NRF-1/GAPDH ratio normalized to control PC12-ND6 cells. Mean ± SEM (*n* = 3; *p ≤ 0.05). **f** Left panel illustrating the quantitative immunoblot analysis of PGC1-α in control PC12 and PC12-ND6 cells as well as NaBt-treated PC12-ND6 cells. Right panel showing the graphed PGC1-α/GAPDH ratio normalized to control PC12 cells. Mean ± SEM (*n* = 3)

In view of NaBt-mediated increase in mitochondrial population, we hypothesized that NaBt may induce mitochondrial biogenesis via mitochondrial DNA (mt-DNA) metabolism. Live-cell confocal microscopy reveals increased number and size of mt-nucleoids in NaBt-treated cells, while mt-nucleoids are barely detectable in control cells (Fig. 3a). By qPCR, we detected a 2.9-fold increase of mt-DNA copy number in NaBt-treated cells (Fig. 3b), in keeping with the twofold increase in mitochondrial mass. Other pan-HDACis, TSA, VPA, and BML-210, also increased mt-DNA copy number, suggestive of a common mechanism to stimulate mt-DNA metabolism (Fig. 3b).

We examined the expression levels of the nuclear-encoded TFAM protein since its levels are directly proportional to mt-DNA copy number^23^. Immunoblot analysis shows a twofold increase in TFAM levels after 2 days of NaBt treatment, which was confirmed by immunocytochemistry (Fig. 3c, d). Since the nuclear respiratory factor 1 (NRF-1) regulates the *Tfam* promoter activity and maintains mt-DNA copy number in vivo^24,25^, we assessed its expression pattern and found increased NRF-1 levels preceding those of TFAM in NaBt-treated cells (Fig. 3e). Finally, we examined the transcriptional coactivator PGC-1α, a nodal regulator of mitochondrial biogenesis in many cell lineages^26^, and found no increased expression in treated cells (Fig. 3f). This is not surprising given that NaBt in part mimics PGC1α by recruiting HATs to the NRF-1 and Tfam promoters^27^. These results indicate that NaBt induces mitochondrial biogenesis and mt-DNA replication via the NRF-1–TFAM axis.

### The timing of NaBt treatment is critical for potentiating sub-optimal neurotrophic cues

**Fig. 4:**
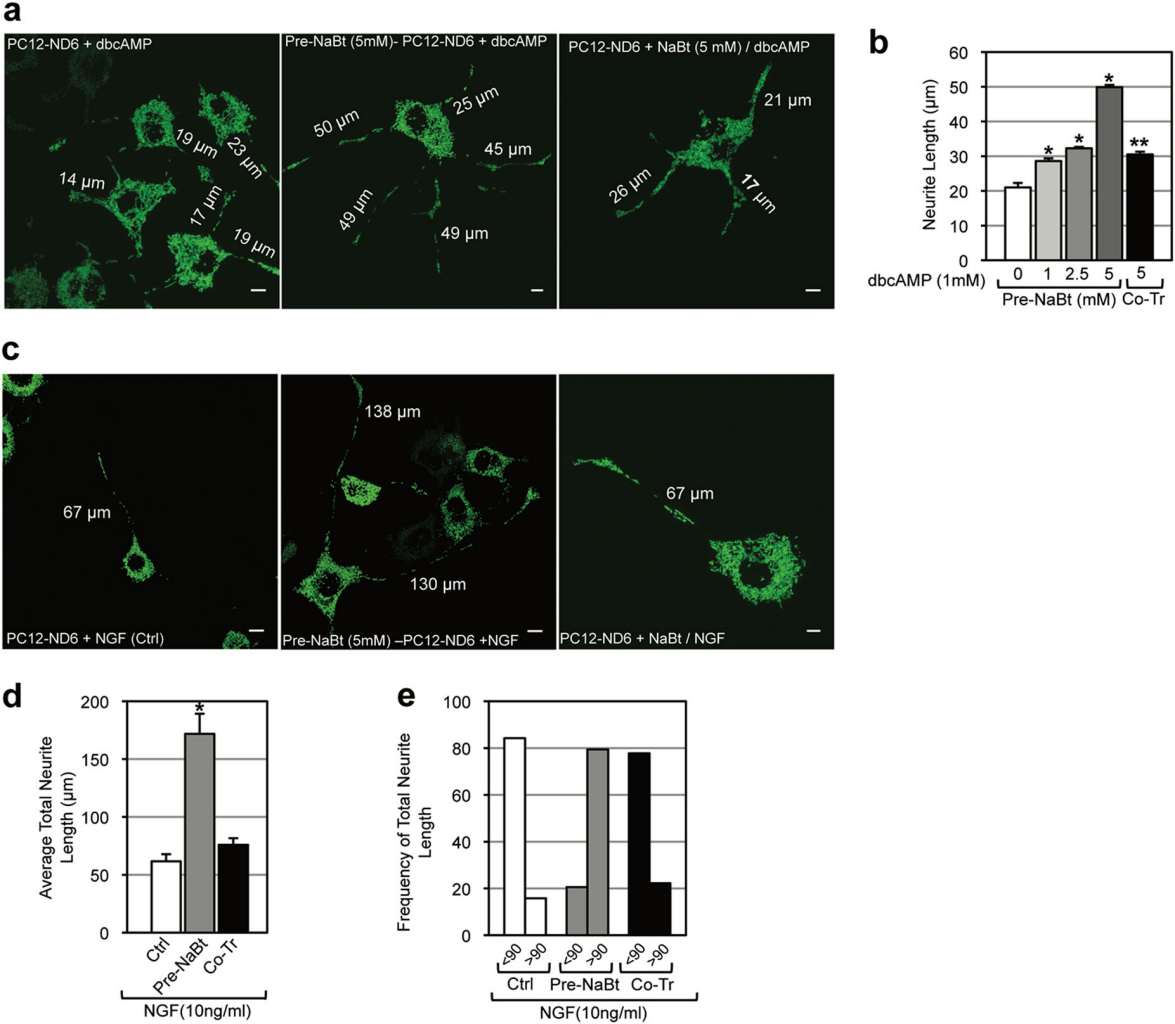
The timing of NaBt treatment is critical for enhancing neuronal differentiation of PC12-ND6 cells upon sub-optimal neurotrophic cues. **a** Representative confocal micrographs of PC12-ND6 cells transfected with the mito-GFP vector and treated with dbcAMP alone (left panel) or with NaBt in a simultaneous (middle panel) or sequential (right panel) manner. Scale bar = 5 µm. **b** Graph of neurite length for each experiemental condition. Mean ± SEM (*n* = 50 for each condition; compared to control; *p ≤ 0.0001). **c** Representative confocal micrographs of PC12-ND6 cells transfected with the mito-GFP vector and treated with sub-optimal NGF concentration (10 ng/ml) alone (left panel) or first NaBt (5 mM) in a simultaneous (middle panel) or sequential (right panel) manner. Scale bar = 10 µm. **d** Graph of average total neurite length for each experimental condition. Mean ± SEM (*n* = 50 for each condition; compared to control; *p ≤ 0.0001). **e** Graph of the frequency of total neurite length below and above 90 µm for each experimental condition

Given that NaBt-stimulated mitochondrial biogenesis and bioenergetics, we explored whether NaBt could potentiate sub-optimal neurotrophic cues in PC12-ND6 cells and whether a temporal window for NaBt responsiveness could be critical to elicit effective neurite outgrowth. We used the dbcAMP experimental paradigm, since it elicits limited neuritogenesis in PC12 cells^21,28^. Pre-NaBt treatment potentiated the neuritogenic activity of dbcAMP in a dose-dependent manner (Fig. 4a, b) by promoting extensive neurite outgrowth after 5 days of dbcAMP exposure, a response that dbcAMP alone could not trigger (Fig. S4A). In contrast, co-treatment with NaBt and dbcAMP severely dampened the NaBt effect on increased neuritogenesis (Fig. 4a, b), suggesting that acquisition of adequate mitochondrial mass precedes onset of neuritogenesis.

We examined the NaBt effect on PC12-ND6 neuronal competency using sub-optimal NGF concentration. Pre-NaBt-treated cells showed accelerated neuritogenesis, whereas control cells displayed a reduced neuritogenesis (Fig. 4c, d). Pre-NaBt treatment also altered the frequency of maximal neurite length triggered by NGF (Fig. 4e), an effect maintained for 3 days (Fig. S4B). No synergistic effect was detected in co-treated cells (Fig. 4e), in keeping with the curtailed effect of co-administering NaBt and dbcAMP (Fig. 4b). Thus, our results indicate that the timing of NaBt exposure is critical to potentiate sub-optimal levels of neurotrophic factors.

### NaBt promotes the acquisition of a critical pool of mitochondria to ensure efficient neuritogenesis

**Fig. 5:**
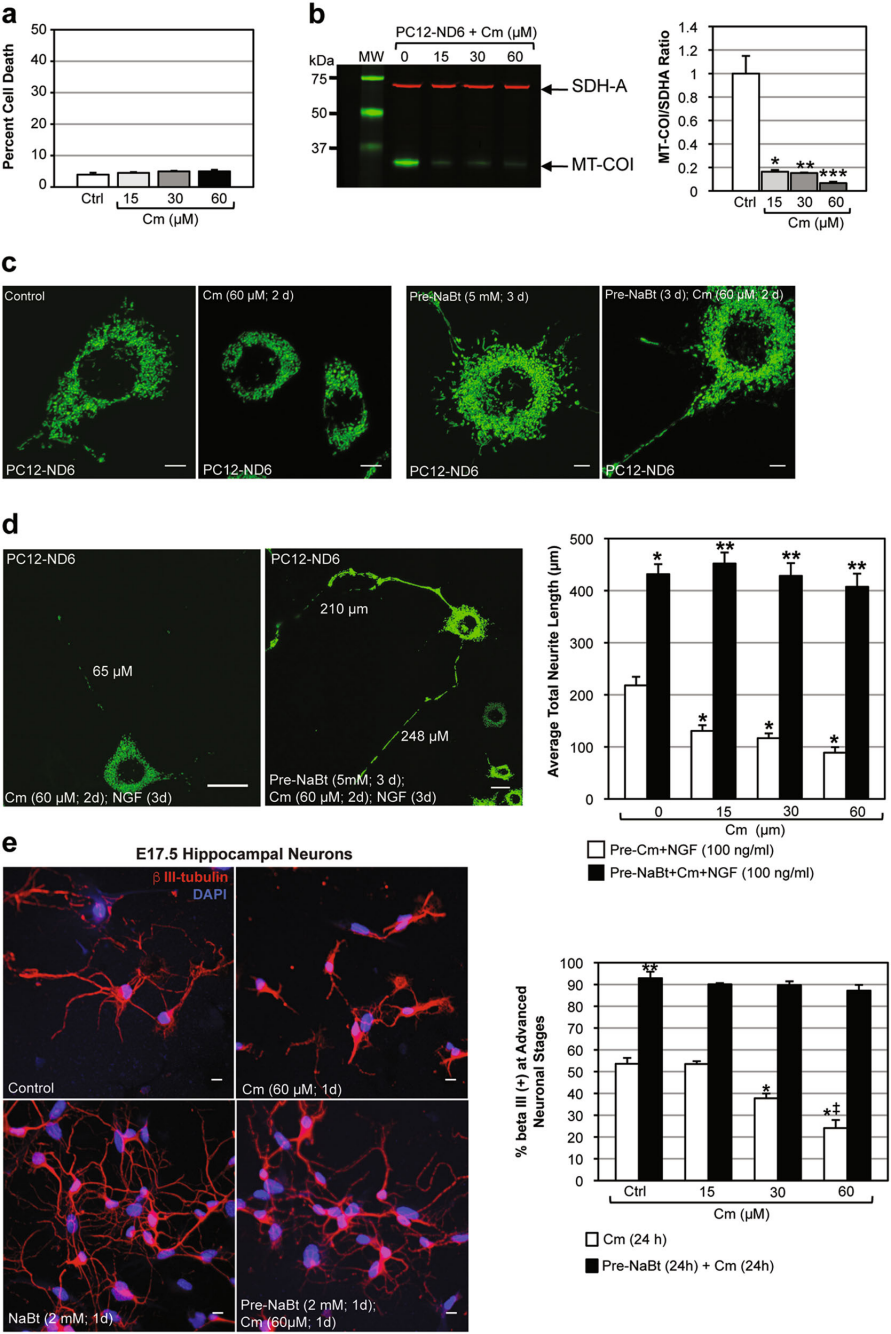
Pre-treatment of PC12-ND6 with NaBt cells negates the inhibitory effect of chloramphenicol on neuronal differentiation. **a** Quantification of PC12-ND6 cell death upon exposure to different concentrations of chloramphenicol (Cm). Data are expressed as percent of cell death ± SEM (*n *= 250 cells). **b** Left panel showing quantitative immunoblot analysis of mitochondrial-encoded MT-COI protein (green) and nuclear-encoded SDH-A protein (red) in untreated and Cm-treated PC12-ND6 cells. Right panel showing quantification of the ratio MT-COI/SDH-A normalized to untreated PC12-ND6 cells. Data are expressed as mean ± SEM (*n* = 3; compared to untreated cells; *p = 0.0052; **p = 0.0048; ***p = 0.0034). **c** Representative live confocal micrographs of untreated and Cm-treated PC12-ND6 cells transfected with the vector mito-GFP (left panels) and of NaBt pre-treated PC12-ND76 cells transfected with the vector mito-GFP prior to vehicle or Cm exposure (right panel). Scale bar = 5 µm. **d** Representative live confocal micrographs of PC12-ND6 cells transfected with the vector mito-GFP and treated with Cm (2 d) followed by NGF exposure for 3 days (left panel) and NaBt pre-treated PC12-ND6 cells transfected with the vector mito-GFP before being exposed to Cm (2 d) followed by 3 days of NGF treatment (middle panel). Scale bar = 20 µm. Right panel showing quantification of average total neurite length for each experimental conditions. Data are expressed in mean ± SEM (*n* = 50 cells; compared to untreated PC12-ND6 cells *p = 0.0001; compared to non-NaBt-treated PC12-ND6 cells with corresponding Cm concentration **p = 0.0001). **e** The left panel shows representative confocal micrographs of E17.5 hippocampal neurons in the absence or presence of Cm (60 µM) at 2 DIV and labeled with anti β-III tubulin antibody (red) and nuclear counterstain DAPI (blue) at 3 DIV (top row) and of confocal micrographs of e17.5 hippocampal neurons treated with NaBt (2 mM) at 1 DIV prior to exposure to Cm (60 µM) at 2 DIV and labeled with anti β-III tubulin antibody (red) and nuclear counterstain DAPI (blue) at 3 DIV (bottom row). Scale bar represents 10 µm. The right panel shows the quantification of E17.5 β-III^+^ hippocampal neurons at the neuronal stage 3. Data are expressed as mean ± S.D. (*n* = 250 cells; compared to untreated control *p = 0.0001; compared to the corresponding non-NaBt-treated sample **p = 0.0001; compared to non-NaBt-treated cells exposed to Cm at 30 µM ^‡^*p* = 0.007)

In view of the temporal window of NaBt-mediated responsiveness to neurotrophic factors, we hypothesized that induction of mitochondrial biogenesis may be a critical step of the neuronal differentiation program. We modulated the mitochondrial mass by blocking or stimulating mitochondrial biogenesis with chloramphenicol (Cm) or NaBt, respectively. Cm selectively inhibits mitochondrial translation without interfering with translation of nuclear-encoded proteins^29^. We first confirmed that Cm did not affect cell viability (Fig. 5a). To assess the efficacy of Cm treatment, we measured the expression levels of the nuclear-encoded succinate dehydrogenase (SDH-A) subunit of Complex II and the mitochondrial-encoded subunit I of Complex IV (MT-COI). While SDH-A expression remained unaltered by Cm, MT-COI expression was abolished at the lowest Cm dose (Fig. 5b). Live-cell confocal microscopy confirms decreased mitochondrial mass in Cm-treated cells, while pre-NaBt-treated cells sustain their mitochondrial biomass even at high Cm doses (Fig. 5c).

To test whether a critical mitochondrial mass is required for optimal neuritogenesis, we treated PC12-ND6 cells with NaBt before a 2-day Cm treatment followed by a 3-day NGF exposure. Low Cm dose reduced total neurite length by 43%, while pre-NaBt treatment optimized neuritogenesis with a sustained mitochondrial density despite high Cm concentration (Fig. 5d). Similarly, Cm treatment of E17.5 hippocampal neurons without NaBt pre-exposure hampered the frequency of stage 3, while NaBt pre-treatment insured a normal progression to stage 3 (Fig. 5e). Thus, acquisition of a critical pool of mitochondrial mediated by NaBt prior to neurotrophic cues is a fundamental step that dictates the efficiency of neuritogenesis and progression of neuronal differentiation. Mitochondrial biogenesis is an integral component of the epigenetic-induced neuronal differentiation program.

### The HAT CBP modulates the mitochondrial biomass in PC12-ND6 cells

**Fig. 6:**
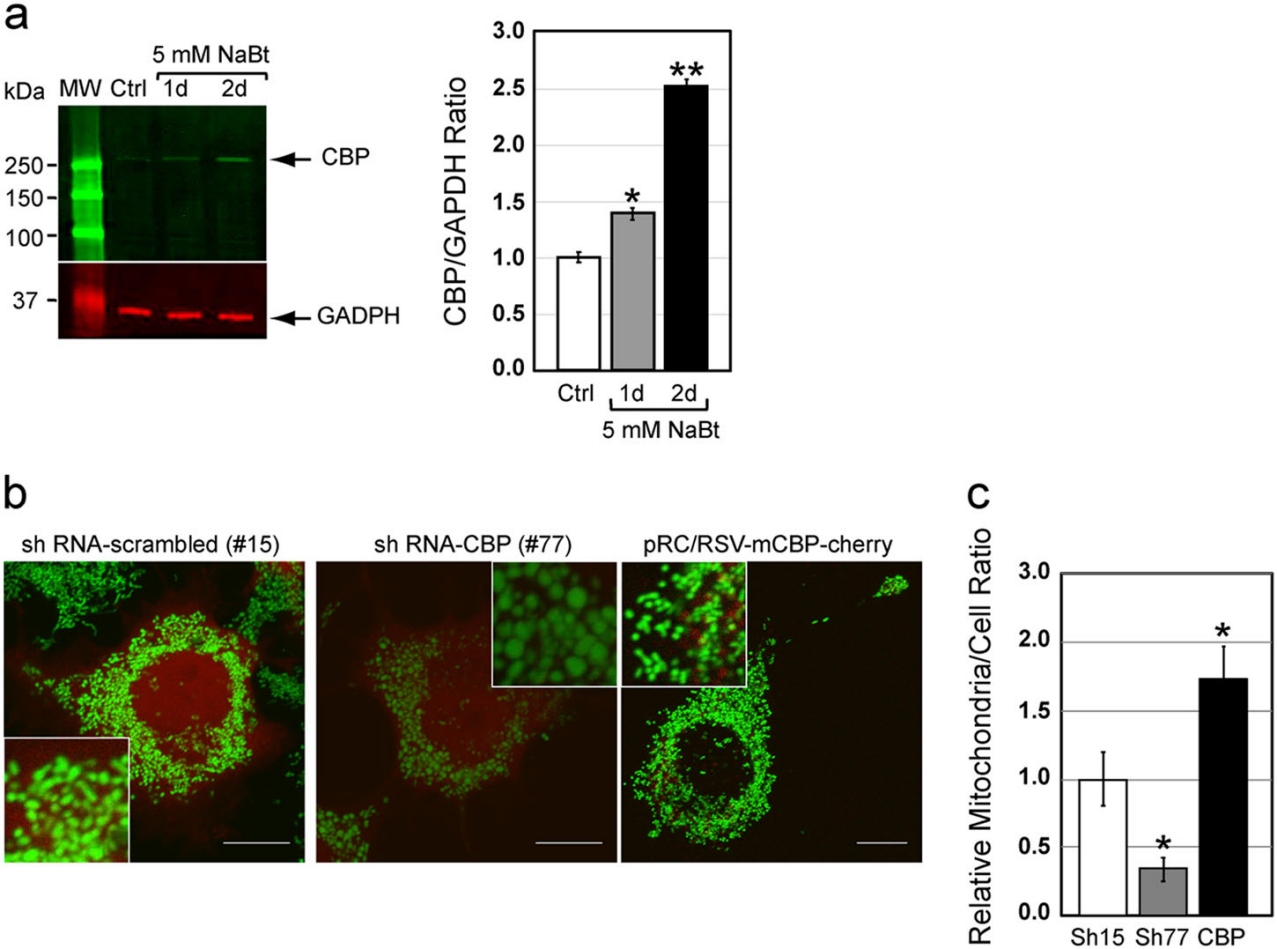
The HAT CBP modulates the mitochondrial biomass in PC12-ND6 cells. **a** Quantitative immunoblot analysis of CBP expression in control and NaBt-treated PC12-ND6 cells. Lower panel showing the graphed CBP/GAPDH ratio normalized to untreated PC12-ND6 cells and presented as mean ± SEM (*n* = 3; *p = 0.04; **p = 0.0001). **b** Representative live confocal micrographs of PC12-ND6 cells transfected with the pRFP-C-RS ratSH shRNA turboRFP vector containining either the negative control shRNA-scrambled #15, specific shRNA-CBP #77, or the CBP overexpressing pRc/RSV-mCBP-HA vector before staining with the MTG dye (scale bar = 10 µm). The insets show a high magnification of mitochondria to illustrate their morphology for each condition. **c** Quantification of mitochondrial integrated density per cell area using stacked confocal images and Image J. Graphed data are presented as mean ± SEM (*n *= 3 experiments for each condition; *p ≤ 0.05)

Since the chromatin modifier CBP regulates differentiation of neuronal precursors via its HAT activity^2^, we investigated whether CBP could modulate the mitochondrial biomass of PC12-ND6 cells. We showed that NaBt increased the levels of CBP expression by a 2.5-fold in PC12-ND6 cells (Fig. 6a), consistent with TSA-mediated rescue of CBP-knockdown in precursor neurons^2^. We knockdowned *cbp* expression using shRNA to assess its effect on the mitochondrial population. We detected a 2.8-fold decrease of the mitochondrial population and an altered mitochondrial morphology with a preponderance of swollen mitochondria, suggesting defective mitochondrial dynamics (Fig. 6b, c). Conversely, CBP overexpression augmented the mitochondrial population by twofold (Fig. 6b, c), comparable to that of the NaBt treatment (Fig. 1b). Thus, our genetic-based analysis confirms our NaBt results and shows that CBP modulates the mitochondrial biomass in PC12-ND6 cells.

### NaBt generates an epigenetic signature in complementary nodes of nuclear-encoded genes regulating neuronal differentiation and mitochondrial reprogramming

**Fig. 7:**
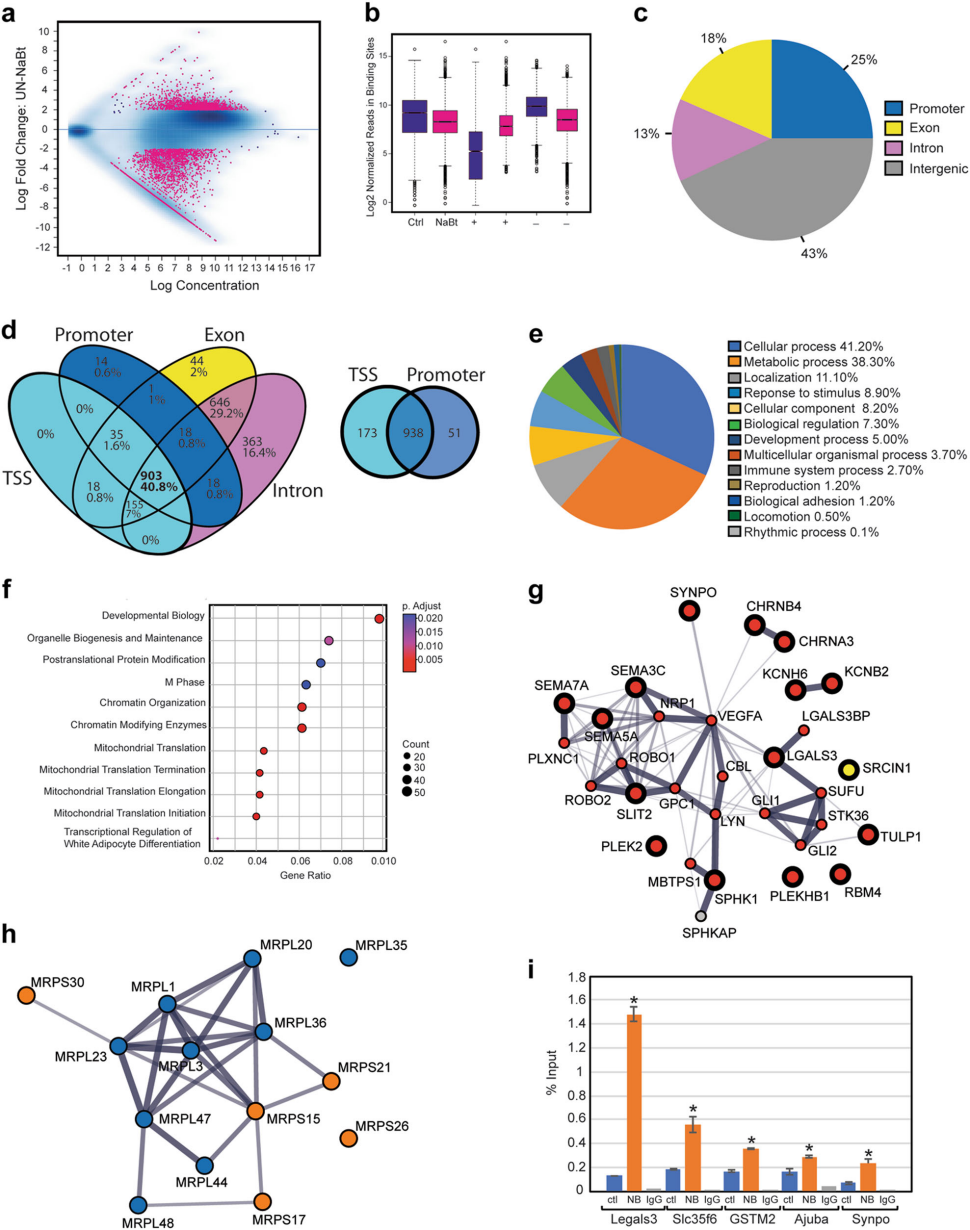
Genome-wide analysis of H3K27 acetylation in control and NaBt-treated PC12-ND6 cells. **a** MA plot of control (untreated) and NaBt-treated PC12-ND6 cells The 3857 differential bound (DB) sites are shown in red (FDR < 0.050). **b** Box plots of read distributions for significantly DB sites. The left two boxes show distribution of reads over all DB sites in the control and NaBt-treated groups; the middle two boxes show distributions of reads in DB sites that increase in affinity in the NaBt group ( + ); the last two boxes show distributions of reads in DB sites that increase in affinity in the control group (−). **c** Distribution of DB H3K27ac sites with respect to intragenic regions including promoter, introns, and exons, and intergenic regions. **d** Venn diagram of unique and overlapping DB H3K27ac sites in different genomic regions. **e** Gene ontology analysis by PANTHER with percent distribution of the DB sites in the identified biological processes. **f** Dot-plot graph of the Reactome pathway enrichment analysis for the DB sites with the corresponding p-values and count indicated for each pathway. **g** In silico networking among the 15 identified neuronal genes with high H3K27ac levels using STRING database. The red circles with thick black borders identify genes from the ChIP-seq analysis with NaBt-mediated increased levels of H3K27ac that are integrated within three neuronal GO processes: axon guidance, signal transduction, and cellular differentiation, while the yellow circles indicate genes with increased H3K27ac that are not interconnected within these three GO processes. The red circles indicate members of this neuronal pathway that were not revealed by the ChIP-seq analysis. The thickness of connectors represents the edge confidence of a given interaction, with a minimum score of 0.9 for thick lines, a minimum score of 0.70 for medium lines, and a minimum of 0.4 score for light lines. **h** In silico networking among the 14 identified MRPs with high H3K27ac levels using STRING database. The blue and orange circles illustrate the small and large subunits of MRPs, respectively. The thickness of connectors represents the edge confidence of a given interaction, as described in **g**. **i** ChIP-qPCR validation of the six genes exhibiting enriched H3K27ac in the presence of NaBt using either a H3K27ac-specific antibody or immunoglobulin G control (gray) and ChIP reactions from control (red) or NaBt-treated PC12-ND6 (blue) cells. Data are expressed in % input and mean ± S.D. from *n* = 2. *p < 0.05

We performed ChIP assays followed by genome-wide deep-sequencing (ChIP-seq) by interrogating the H3K27ac signature, a hallmark of active enhancers. We opted for a 3-day NaBt treatment to concomitantly capture gene networks controlling neuronal differentiation and mitochondrial reprogramming. ChIP-seq assays were performed on two independent replicates from control and NaBt-treated cells. The H3K27ac peaksets were highly reproducible between biological replicates, with the PCA analysis confirming the existence of two separate groups of differential binding (DB) sites (Fig. S5). The MA plot indicates 3857 DB sites with at least a two-fold change and a FDR < 0.05, 2281 DB in NaBt-treated cells and 1548 in control cells (Fig. 7a). The box plots of read distributions confirms increased affinity in NaBt-treated cells when comparing the higher median in the ( + ) column of treated cells with that of the (−) column of control cells (Fig. 7b). 25% of DB sites mapped in promoter regions, while 31 and 43% of DB sites were located within genic and intergenic regions, respectively (Fig. 7c). Using Venny^30^ on DB sites with at least a two-fold change, a *p*-value < 0.05, and a FDR < 0.05, we found that nearly 41% of the DB sites mapped in the TSS, promoter, exons and introns regions of 903 genes, while very few peaks were specific to a single genomic region (Fig. 7d). The promoter region was defined as ±1 Kbp of the TSS, while the TSS region was demarcated by a peak overlapping the actual TSS. Given the considerable overlap between these two annotations (Fig. 7d), subsequent analyses were performed on the 1099 DB sites mapping in 989 promoter regions (Table S1).

Using PANTHER^31^, gene ontology analysis reveals gene enrichment in the GO cellular and metabolic processes, which share genes involved in neurotrophic response, chromatin organization, mitochondrial homeostasis, and energy metabolism (Fig. 7e). Similarly, the reactome pathway analysis shows several nodes for developmental biology, organelle biogenesis, chromatin organization, and mitochondrial translation with significant p-values and gene counts (Fig. 7f). Using STRING database, we identified enriched DB sites in many neuronal genes, 15 of those involved in axon guidance with high level of connectivity (Fig. 7g). It also reveals enrichment in 14 genes encoding mitochondrial ribosomal proteins (MRPs) (Fig. 6h), which are integral components of mitochondrial ribosomes responsible for translation of the 13 mitochondrial-encoded OXPHOS proteins^32^. Except for the MRPL36 promoter, the ChIP-seq results indicate a slight decrease in number of reads for 13 MRPs in NaBt-treated cells (Table S1). This trend is consistent with our immunoblot kinetic results showing increased expression of the NRF-1 and TFAM regulators of mitochondrial biogenesis, during its initial surge during the first 2 days of NaBt treatment, whereas its maintenance more likely occurs at day 3 (Fig. 3c, f). Among the 1099 DB sites, we found 55 DB sites exhibiting a unique H3K27ac signature with high amounts of reads in NaBt-treated cells and barely any reads in control cells, all of them involved in cytoskeletal remodeling, synaptic activity and mitochondrial metabolism (Table S2). Our ChIP-qPCR validation analysis on five of those genes, *Ajuba*, *Lgals3*, *Synpo*, Slc35f6, and *Gstm2*, shows NaBt-mediated enrichment of H3K27ac mapping in their promoters in accordance with the ChIP-seq results (Fig. 7i).

## Discussion

In this study, we report a novel developmental epigenetic layer that couples mitochondrial biogenesis to neuronal differentiation. Modulation of the mitochondrial biomass using gain- and loss-of-function-like pharmacological assays has exposed the timing of mitochondrial biogenesis as a key event to enhance neuronal progenitor competency to differentiate. Our collective results demonstrate that epigenetic regulation of mitochondrial reprogramming is an integral component of the overall epigenetic signature induced by neurotrophic signaling in preparation for upcoming energy needs associated with neuronal differentiation. CBP regulates the mitochondrial biomass, congruent with its role as a regulator of neural precursor competency via its HAT activity^2^. Given that half of the mitochondrial proteome is tissue-specific^33^, epigenetic regulation of mitochondrial biogenesis and bioenergetics provides a mechanistic layer to tailor and synchronize the mitochondrial proteome to a specific cellular and developmental context. NaBt confers an H3K27ac epigenetic signature in several nodes of nuclear genes promoting neuronal differentiation and mitochondrial reprogramming. Our findings add a new facet to the mechanistic understanding of how pan-HDACis induce differentiation of neuronal progenitor cells, thereby extending previous studies that only characterized the molecular events coordinating neuronal differentiation to cell cycle exit via upregulation of NeuroD and the CDKi p21^WAF/cip1^^11,12^. Increased mt-DNA replication by four pan-HDACis suggests a common mechanism by which they regulate mitochondrial biogenesis.

Until recently, little emphasis has been placed on the neuronal-specific regulation of mitochondrial biogenesis. We previously reported NeuroD6 promoting mitochondrial biogenesis at the outset of neuronal differentiation via the NRF-1/TFAM axis^15–17^. Increased mitochondrial biogenesis via Tfam expression was also reported in ES cells and progenitor-like cells undergoing neuronal differentiation upon retinoic acid treatment^34,35^. Exposure of embryonic hippocampal neurons to the morphogen Sonic Hedgehog increases mitochondrial mass during axonal outgrowth^36,37^, while activation of mitochondrial biogenesis in embryonic cortical neurons coincides with neuritogenesis^38,39^.

Our findings extend those previous studies by identifying a novel epigenetic regulatory layer that synchronizes the timing of mitochondrial reprograming with neurotrophin-induced differentiation. Our gain-and loss-of-function results reveal mitochondrial biogenesis as a critical preparatory step for induction of differentiation. NaBt-mediated increase of TFAM expression and mt-DNA copy number is physiologically pertinent, as TFAM is a limiting determinant of mt-DNA copy number and essential for in vivo mitochondrial biogenesis. While embryonic disruption of the *Tfam* gene triggers early embryonic lethality due to a lack of mt-DNA and severe OXPHOS deficiency, *Tfam* postnatal disruption in hippocampal neurons decreases mt-DNA copy number and OXPHOS capacity leading to axonal degeneration^23,40^. Conversely, *Tfam* transgenic mice harbor increased mt-DNA copy number^41^.

Epigenetic regulation of mitochondrial reprogramming is congruent with the requirement of a specific landscape of histone acetylation for neuronal lineage progression^1,2,4^. Our results evoke a NeuroD6–CBP interplay coordinating epigenetic status, mitochondrial biogenesis and neuronal differentiation^15,16^. NeuroD6 expression is regulated by the transcription factor C/EBPβ, which favors neurogenesis by sequestering CBP from STAT to activate neuronal genes^42,43^. Our Ajuba ChIP-seq and ChIP-qPCR results substantiate our model of epigenetic regulation synchronizing neuronal differentiation and mitochondrial reprogramming. Ajuba increases histone acetylation by recruiting CBP to PPARγ, thereby activating expression of PPARγ target genes, such as the C/EBP transcription factors^44^. Thus, the Ajuba–CEBP interconnection can be viewed as a component of the NeuroD6–CBP axis adapting mitochondrial biogenesis to neuronal differentiation^15,17,42^.

Moreover, NaBt induces an H3K27ac epigenetic fingerprint in the MRPs node, which is critical for mitochondrial biogenesis via their role in translation of mitochondria-encoded OXPHOS subunits^15,17,45^. It is clinically relevant, as mitochondrial respiratory neurodevelopmental disorders exhibit defective mitochondrial biogenesis, mitochondrial protein synthesis, and OXPHOS^46^. Mutations in the *MRPL16*, *MRPL3*, and *MRPL12* genes cause corpus callosum agenesia, psychomotor retardation, and neurological deterioration, respectively^47,48^.

The validated genes, *Lgals3* and *synaptopodin*, promote axonal and dendritic outgrowth, respectively, which depend on mitochondrial homeostasis. In hippocampal and dorsal root ganglion neurons, Lgals3 induces axonal branching via enrichment of F-actin branchlets at the site of protrusive activity, a process requiring local stalling of mitochondria and increased OXPHOS activity^49,50^. The actin-binding protein synaptopodin modulates synaptic activity of cortical and hippocampal neurons by promoting formation of dendritic spines and regulating local Ca^2+^ levels, processes in which mitochondria are critical^51,52^.

Epigenetic regulation also coordinates changes in mitochondrial ultrastructure with enhanced OXPHOS metabolism. This is particularly relevant for mitochondrial biogenesis, which requires maintenance of the mitochondrial membrane potential for importing nuclear-encoded proteins involved in diverse mitochondrial functions, such as OXPHOS, mtDNA replication and transcription, mitochondrial dynamics and membrane biogenesis^46^. The validated *Slc35f6* gene stimulates ΔΨ_m_ by interacting with the mitochondrial ATP transporter ANT2 embedded in the mitochondrial inner membrane to transport ATP out to the cytosol while importing ADP into the mitochondrial matrix^53,54^. Our ChIP-seq results corroborate the NeuroD6-mediated synchronization of enhanced ΔΨ_m_ and mitochondrial biogenesis with onset of neuronal differentiation^17^, which was also reported in neuroblasts and axons of NGF-treated sensory neurons^55,56^.

Epigenetic regulation calibrates metabolic functions to match high energetic demand during the onset of neuronal differentiation by increasing maximal mitochondrial respiration activity and generating considerable spare respiratory capacity to confer resistance to oxidative stress and excitotoxicity during differentiation. This is consistent with NeuroD6 mitigating ROS levels generated by increased OXPHOS activities in neuronal precursors, and increased mitochondrial bioenergetics in embryonic cortical neurons and neural progenitor cells derived from iPSCs^17,38,57,58^.

**Fig. 8:**
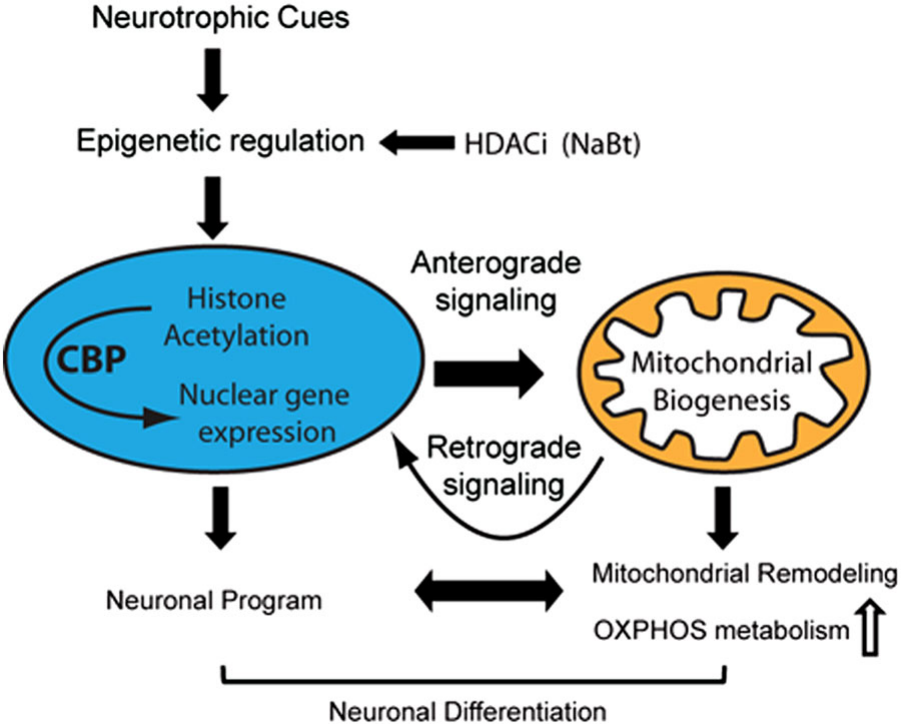
Working model on the coordinated epigenetic regulation of mitochondrial biogenesis and neuronal differentiation. The HDACi NaBt in part recapitulates the epigenetic regulation induced by neurotrophic signaling in which the CBP chromatin modifier regulates the mitochondrial mass in differentiating neuronal precursor cells. Epigenetic regulation modulates the mitonuclear crosstalk via the anterograde and retrograde sinaling to synchronize the two distinct genomes

In summary, our findings lend support to our working model whereby epigenetic regulation dynamically synchronizes the mitochondrial and neuronal programs for efficient neuronal differentiation (Fig. 8). NaBt in part recapitulates neurotrophic signaling by generating an H3K27ac signature promoting these two programs, with CBP playing a key role in modulating the mitochondrial mass. Epigenetic regulation also influences the mitochondrial–nuclear crosstalk by coordinating gene expression from these two distinct genomes via anterograde and retrograde signaling with mitochondria producing the acetyl moiety for histone acetylation of nuclear genes. Future studies will elucidate the HDACis-mediated comprehensive epigenetic signature of these programs to set the stage for investigations in the context of metabolic and neurodegenerative diseases with mitochondrial dysfunctions.

## Materials and methods

### Preparation of embryonic E17.5 hippocampal neurons and transfection

E17.5 hippocampal neuron cultures were prepared as described^59^. All animal procedures were carried in compliance with the Institutional Animal Care and Use Committee. Briefly, hippocampi were dissected from E17.5 mouse fetuses in ice-cold Hanks balanced salt solution (HBSS) modified (Hyclone). Isolated hippocampi were digested with 20 U/ml of papain (Worthington) and 2000 U/ml DNase1 (Worthington) at 37 °C for 60 min before being triturated with a 10 ml pipette. Cells were centrifuged at 900 rpm for 5 min and washed with HBSS. Neurons were resuspended in DMEM supplemented with 10% fetal bovine serum and penicillin/streptomycin before being plated at a concentration of 3 × 10^5^ cells on 35 mm^2^ glass-bottom plates (Warners Instruments) coated with 0.01% poly-l-ornithine (Sigma). In experiments using neurons transfected with the mito-GFP vector (1 µg; Clontech), the Amaxa nucleofector system was used according to the manufacturer’s recommendations (Lonza) and dissociated neurons were transfected prior to plating, as described above. After incubation in a 5% CO2 incubator at 37 °C overnight, neurons were then switched to Neurobasal medium containing 2 mM glutamine, B27, 10 ng/ml hFGF and penicillin/streptomycin. All reagents were purchased from Invitrogen, unless stated otherwise.

### PC12-ND6 cell culture and transfection

PC12-ND6 cells were grown and transfected with the mito-GFP or mito-RFP vector or the pRc/RSV-mCBP-HA plasmid (Addgene) by electroporation (BTX, Harvard Apparatus), as described^17^. For shRNA-based silencing of CBP, we transfected PC12-ND6 cells with the pRFP-C-RS ratSH shRNA turboRFP vectors (Origene, Rockville, MD containing either the scrambled negative control shRNA cassette (Cat # TR30015) or the rat CBP targeting shRNA FI750477 (AAGACTGTGGAGGTCAAGCCAGGAATGAA) (Cat# TF712619). For immunocytochemistry, cells were grown on poly-d-lysine (PDL)-coated coverslips. For live cell confocal microscopy, cells were seeded onto 50 mm PDL-coated glass bottom plates (Warners Instruments).

### Drug treatment

PC12-ND6 cells and E17.5 hippocampal neurons were exposed to different concentrations (2 mM, 5 mM, or 10 mM) of NaBt for indicated periods of time. PC12-ND6 cells were treated with VPA (1 mM), TSA (600 nM), or BML-210 (10 µM) for 2 days, when indicated. PC12-ND6 cells were treated with dbcAMP (1 mM; Sigma) or NGF (Roche Molecular Biochemicals) at 10 ng/ml or 100 ng/ml. PC12-ND6 cells and E17.5 hippocampal neurons were exposed to different concentrations (15, 30, and 60 µM) of chloramphenicol (Sigma) for the indicated time period. All HDACis, except BML-210 (Enzo Life Sciences), were purchased from Sigma.

### Cell viability assay

Cell viability was assessed by live-cell confocal microscopy using the Nuclear-ID™ Blue/Red viability assay (Enzo Life Sciences) according to the manufacturer’s recommendations. Live cells were identified by blue fluorescence (excitation: 350 nm; emission: 461 nm), whereas dead cells were identified by red fluorescence (excitation: 571 nm; emission: 639 nm). The percent of cell death was quantified using Image J software.

### Quantitative immunoblot analysis

Whole cell extracts were isolated and resolved as described^57^. Nitrocellulose membranes were probed with various primary antibodies (Table S3) overnight, and then incubated for 1 h with anti-mouse or anti-rabbit secondary antibody directly conjugated to a 680-nm or 800-nm fluorescent dye (LI-COR). Samples were simultaneously normalized to an endogenous reference (GADPH). Fluorescence signals were measured and quantified using the Odyssey imaging system (LI-COR).

### Immunocytochemistry

Immunocytochemistry was performed as described^16^ with primary and secondary antibodies listed in Table S3.

### Confocal microscopy and quantification of neurite length

For fixed cells, images were acquired with a ×63 or ×100/1.46 oil immersion objective using the Zeiss LSM 710 confocal microscope. For live-cell confocal microscopy, cells were visualized with a 100×/1.46 oil immersion objective using the Zeiss LSM 510 confocal microscope equipped with a TempModuleS and CO_2_ Module (Zeiss) to maintain the temperature and CO_2_ levels at 37 °C and 5.0%, respectively. Combined differential interference contrast (DIC) and fluorescence confocal imaging were used to measure neurite length in transfected live cells. Imaging was acquired with a *z* stack to ensure that the full length of neurites is acquired. Neurite length was measured on fixed cells immunostained with an anti-β-III tubulin antibody (Table S1). The function measurement of the Zen software (Zeiss) was used and a neurite outgrowth threshold was set at the diameter of cell body.

### Quantification of mitochondria in cultured E17.5 hippocampal neurons

E17.5 hippocampal neurons were grown in the absence or presence of NaBt (2 mM) at 1 DIV, fixed and permeabilized at 3 DIV as described^15^. Differentiating neurons were identified using an anti-βIII tubulin antibody (Table S1) and mitochondria were labeled with an anti-COXVα antibody (Table S1). All cells were counterstained with nuclear stain DAPI. Confocal images were acquired using the Zeiss LSM 710 system and a ×100/1.46 oil immersion objective. Stack images of neuronal cells at stage 3 were captured using the optimal overlapping (50%) option from the Zen software to insure complete stacking on 25 cells from two independent experiments for each experimental condition. Cell and mitochondrial volumes were calculated using Volocity (v5.0) software, as described^53^.

### Labeling of mitochondrial nucleoids

PC12-ND6 cells were first transfected with the mito-RFP vector (Clonetech) by electroporation and cultured in the absence or presence of NaBt (5 mM) for 2 days. Untreated and treated PC12-ND6 cells were incubated in 1 ml of serum-free medium containing 3 µl of the fluorescent DNA dye PicoGreen® (Invitrogen) for 1 h at 37 °C. Cells were washed with PBS and images were acquired by live cell confocal microscopy in the presence of phenol red-free medium using a ×100/1.46 objective.

### Mitochondrial DNA content by quantitative PCR

Total cellular DNA from three independent samples of untreated and NaBt-treated PC12-ND6 cells was isolated by phenol-chloroform extraction using standard methods. Mitochondrial DNA was quantified by normalizing the mitochondrial-encoded gene COXI (mtco1) to the nuclear-encoded gene Ndufv1 using qPCR and the ∆∆CT method. Primers used in this study were: 5′-ACCCCCTGCTATAACCCAATATCAGAC-3′ (F, COXI), 5′-TGGGTGTCCGAAGAATCAAAATAG-3′ (R, COXI), 5′-CGCCATGACTGGAGGTGAGGCAG-3′ (F, Ndufv1), and 5′-GGCCCCGTAAACCCGATGTCTTC-3′ (R, Ndufv1). SYBR Green fluorescence detection for qPCR was performed using iCycler iQ Real-time Detection System (Bio-Rad). A total of 10 ng total DNA was used in a 25 µl reaction containing 1× SYBR Green iCycler iQ mixture and 0.2 µM of forward and reverse gene-specific primers. Data are expressed as mean ± SEM.

### Measurement of mitochondrial mass by flow cytometry

Untreated and NaBt-treated PC12-ND6 cells were stained with either 50 nM Mitotracker® Red (MTR) (chloromethyl-X-rosamine; Molecular Probes) or 70 nM Mitotracker® Green (MTG; Molecular Probes) and analyzed with a FACScalibur flow cytometer (BD Bioscience) as described^15^. Data were collected from 20,000 cells for each sample from three independent experiments.

### Measurement of the mitochondrial membrane potential (ΔΨ_m_)

Cells were incubated with 25 nM TMRM (Invitrogen) at 37 °C for 45 min. ΔΨ_m_ was assessed by live confocal fluorescence microscopy, in the presence of diluted TMRM (5 nM). ΔΨ_m_ was quantified by flow cytometry analysis using a FACScalibur flow cytometer, as described^57^. Data were collected from 20,000 events for each sample per experiment (*n *= 4) and analyzed using CellQuant software. Data were normalized against the TMRM-emitted fluorescence intensity of untreated PC12-ND6 cells ±S.D.

### Live cell measurement of mitochondrial respiration

Bioenergetic status was measured using the Seahorse Extracellular Flux XFp Analyzer (Seahorse Bioscience, Agilent Technologies). PC12-ND6 cells were seeded at a density of 20,000 cells per well in triplicate on poly-d-lysine-coated plates and treated with PBS (control) or NaBt (5 mM) for 24 h at 37 °C in 5% CO2 atmosphere. Prior to the assay, the F12K medium was changed to unbuffered Base Medium (Seahorse Bioscience) supplemented with 2 mM glutamine (Invitrogen), 2 mM pyruvate (Sigma), and 7.1 mM glucose (Sigma) adjusted to at pH 7.4 with NaOH for 1 h at 37 °C. Using the XF Mito Stress Test kit (Seahorse Bioscience), OCR and ECAR were measured under basal conditions and after sequential injections of oligomycin (1 µM final concentration), FCCP (fluoro 3-carbonyl cyanide-methoxyphenyl hydrazine; 1 and 2 µM final concentrations) and a mix of rotenone and antimycin A (1 µM final concentration) following the manufacturer’s recommendations. The data were normalized to cell numbers and plotted as OCR (oxygen consumption rate; pmol/min/cell) and ECAR (extracellular acidification rate; mpH/min/cell) as a function of time.

### Analysis of mitochondrial ultrastructure by transmission electron microscopy

Control and NaBt-treated PC12-ND6 cells were fixed in 2.5% glutaraldehyde (Electron Microscopy Sciences), 1% paraformaldehyde in 0.12 M sodium cacodylate buffer (Electron Microscopy Sciences) for 20 min at room temperature followed by 40 min on ice. Cells were then fixed for 1 h in 1% osmium tetroxide (Electron Microscopy Sciences) followed by en-bloc staining overnight in 1% aqueous uranyl acetate. The cells were then dehydrated through a series of ethyl alcohol/deionized water solutions and propylene oxide before infiltration with Embed812 epoxy resin. Blocks were cured for 48 h at 60 °C. Polymerized blocks were trimmed and 70 nm ultrathin sections were cut with a diamond knife on a Leica Ultramicut EM UC7 and transferred onto 200 mesh copper grids. Sections were counterstained with 1% ethanolic uranyl acetate for 10 min and lead citrate for 2 min. Samples were imaged with a FEI Talos F200X-transmission electron microscope (FEI Company) operating at an accelerating voltage of 80 kV equipped with a Ceta™ 16M camera. For each condition, 20 cells were analyzed and mitochondrial lengths were measured using Image J software.

### Assessment of ROS levels

When indicated, PC12-ND6 cells were treated with NaBt (5 mM) for 48 h before being labeled with 0.5 µg/ml Hoechst 33342 (Invitrogen) and 2.5 µM MitoSOX Red (Invitrogen), as described^57^.

### ChIP-seq assay and analysis of ChIP-seq data

The ChIP-seq assay was performed with two biological replicates per condition. Untreated and NaBt-treated PC12-ND6 cells (6 × 10^6^ per condition) were cross-linked with 1% formaldehyde for 8 min. The recation was stopped glycine (125 mM) for 5 min. Chromatin immunoprecipitation against H3K27Ac was performed by Diagenode ChIP-seq Service (Cat# G02010000), as were subsequent analyses. Briefly, nuclei were extracted using Diagenode iDeal ChIP-seq kit for histone and chromatin was sheared with the Bioruptor® Pico (Diagenode). ChIP assay was performed using 1 µg of anti-H3K27Ac Premium antibody (Diagenode C15410196, lot A1723-0041D), rabbit IgG (C15410206) as a negative control, or H3K4me3 (C154100003, lot 5051-001P) as a positive control. DNA recovered from chromatin that was not immunoprecipated was used as input. To quantify the ChIP-enriched and input DNA, we used primers for TSH2B as a negative locus (Diagenode, Cat# C17031043-50) and for GAPDH as a positive locus (Diagenode, Cat# C17031046-50).

The input and ChIP samples were sequenced by Illumina HiSeq 2500. Quality control of sequencing reads was performed using FAstQC. The 50-nt sequence reads were aligned to the UCSC rat genome rn5 using the GreyListChIP Bioconductor package to identify regions showing anomalously high signals and to filter the rat samples accordingly. The samples were deduplicated using the SAMtools version 1.3.1. Alignment coordinates were converted to BED format using BEDTools v.2.17 and H3K27ac peak calling was performed using SICER v1.1^60^. Differential H3K27ac binding was analyzed using the DiffBind R/Bioconductor package^61^. The GO term enrichment analysis was carried out on differentially bound (DB) sites mapping in the TSS regions with at least a two-fold change and FDR < 0.05 using the topGO R/Bioconductor package and data from the org.Rn.eg.db package^62,63^. Biological pathways of DB sites H3K27ac were analyzed using ReactomePA and the Reactome Pathway Knowledgebase^64,65^. The degree of connectivity among the neuronal and MRPs proteins was analyzed using STRING version 10.0 with the following parameters: textmining, experiments, databases, co-expression and co-occurrence. The MRPs network was built using an edge confidence between 0.4 and 0.9. The neuronal network with no more than ten interactors for first shell and five interactors for second shell. The candidates neuronal genes belong to axon guidance (FDR = 1.8 × 10^−8^), signal transduction (FDR = 2.38 × 10^−5^), and cellular differentiation (FDR = 1.78 × 10^−3^).

### ChIP-qPCR

ChIP was performed as described above. qPCR primer sequences are listed in Table S4. A standard curve of input DNA was run for each primer set, and the amplification efficiency, the melting profile and the dimerization of the primers (amplification in non-template control) were calculated. qPCR was performed in duplicate using Realtime qPCR (Sybr green) on ChIP DNA with LC96 (Roche). Data are represented as % input ± SEM.

### Statistical analysis

Statistical analyses were performed using the unpaired Student's *t*-test, with *p*-value of less than 0.05 considered statistically significant. Data are expressed as mean ± S.D. unless indicated otherwise.

## Electronic supplementary material

Supplementary figures S1 to S5 including figure legends

Table S1

Table S2

Revised Table S3

Table S4
