# Supplementary material for: Epigenetic modifiers promote mitochondrial biogenesis and oxidative metabolism leading to enhanced differentiation of neuroprogenitor cells
Source: Cell Death Dis. 2018 Mar 2;9(3):360. doi: 10.1038/s41419-018-0396-1 (PMC5834638; doi:10.1038/s41419-018-0396-1)
Supplement: Supplementary file 1 — Supplementary figures S1 to S5 including figure legends [file 41419_2018_396_MOESM1_ESM.docx]

**Supplemental materials:**

**
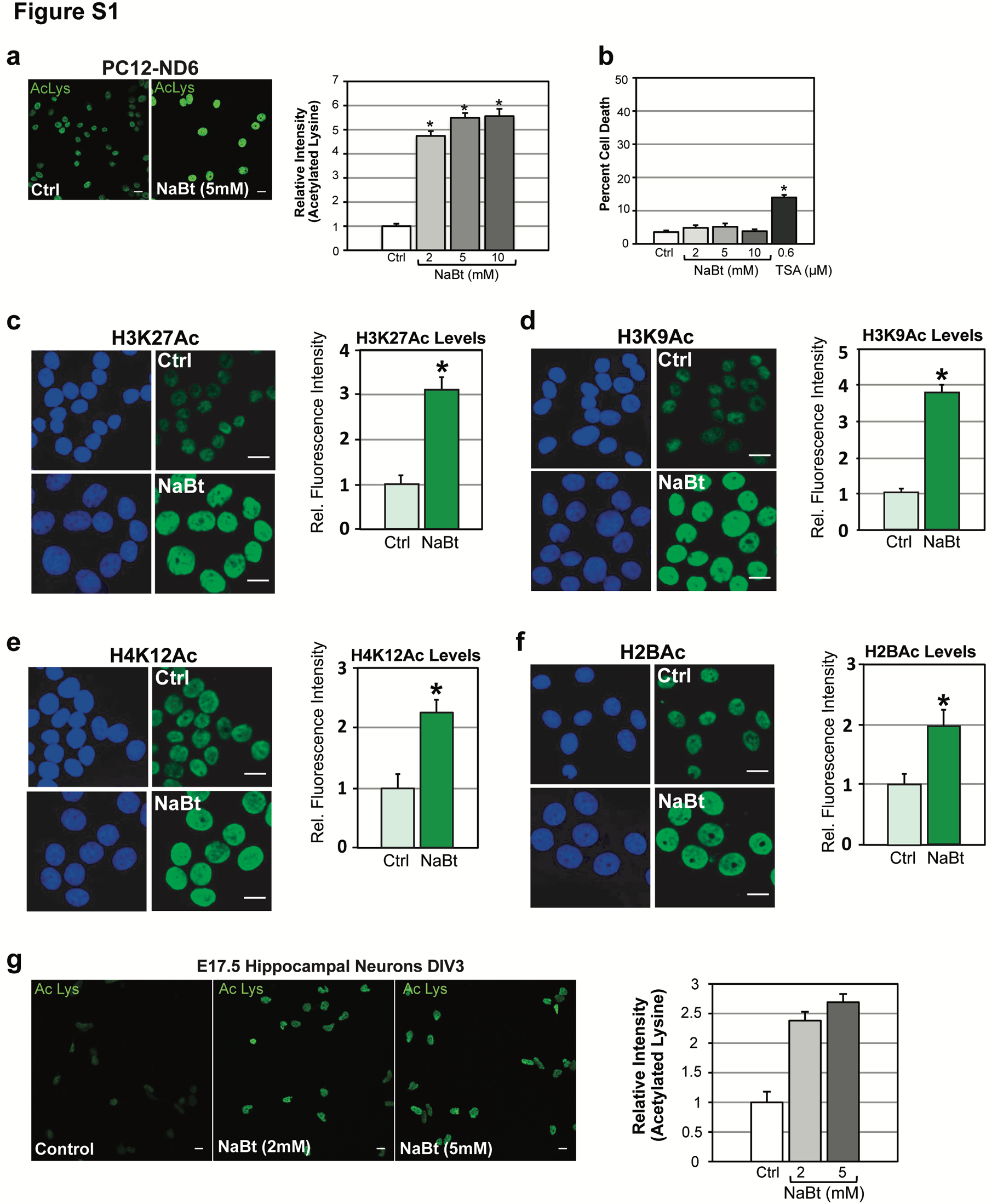
**

**Supplementary Figure 1:** Acetylation response mediated by sodium butyrate. (**a**) Left panel showing representative confocal micrographs of control and NaBt-treated PC12-ND6 cells labeled with an anti-acetylated Lys antibody (AcLys; green). Scale bar = 10 µm. Right panel showing quantification of the NaBt concentration-dependent acetylation of nuclear proteins in control and NaBt-treated PC12-ND6 cells. Data are expressed as relative fluorescence intensity ± S.D. (n=250 cells per NaBt concentration; * p=0.0001). (**b**) Effect of NaBt on PC12-ND6 cell viability. As a control, PC12-ND6 cells were treated with the HDAC inhibitor TSA (0.66 µM) known to induce mild cell death. Data are expressed as mean ± S.D. (n=250 cells for each experimental condition; * p=0.0001). (**c**) Left panel showing representative confocal micrographs of control and PC12-ND6 cells labeled with an anti-acetylated H3K27 antibody. Scale bar = 10 µm. Right panel showing quantification of H3K24ac levels in control and NaBt-treated PC12-ND6 cells. (**d**) Left panel showing representative confocal micrographs of control and PC12-ND6 cells labeled with an anti-acetylated H3K9 antibody. Scale bar = 10 µm. Right panel showing quantification of H3K9ac levels in control and NaBt-treated PC12-ND6 cells. (**e**) Left panel showing representative confocal micrographs of control and PC12-ND6 cells labeled with an anti-acetylated H4K12 antibody. Scale bar = 10 µm. Right panel showing quantification of H4K12ac levels in control and NaBt-treated PC12-ND6 cells. (**f**) Left panel showing representative confocal micrographs of control and PC12-ND6 cells labeled with an anti-acetylated H2B antibody. Scale bar = 10 µm. Right panel showing quantification of H2Bac levels in control and NaBt-treated PC12-ND6 cells. Data in panels c-f are expressed as mean mean ± S.D. (n=250 cells for each experimental condition; * p=0.0001). (**g**) Left panel showing representative confocal micrographs of NaBt dose-dependent acetylation in E17.5 hippocampal neurons (AcLys; green). Scale bar represents 10 µm. Right panel showing quantification of acetylated lysine residues. Data are expressed as relative fluorescence intensity ± S.D. (n=250 cells per NaBt concentration; *p=0.0001).


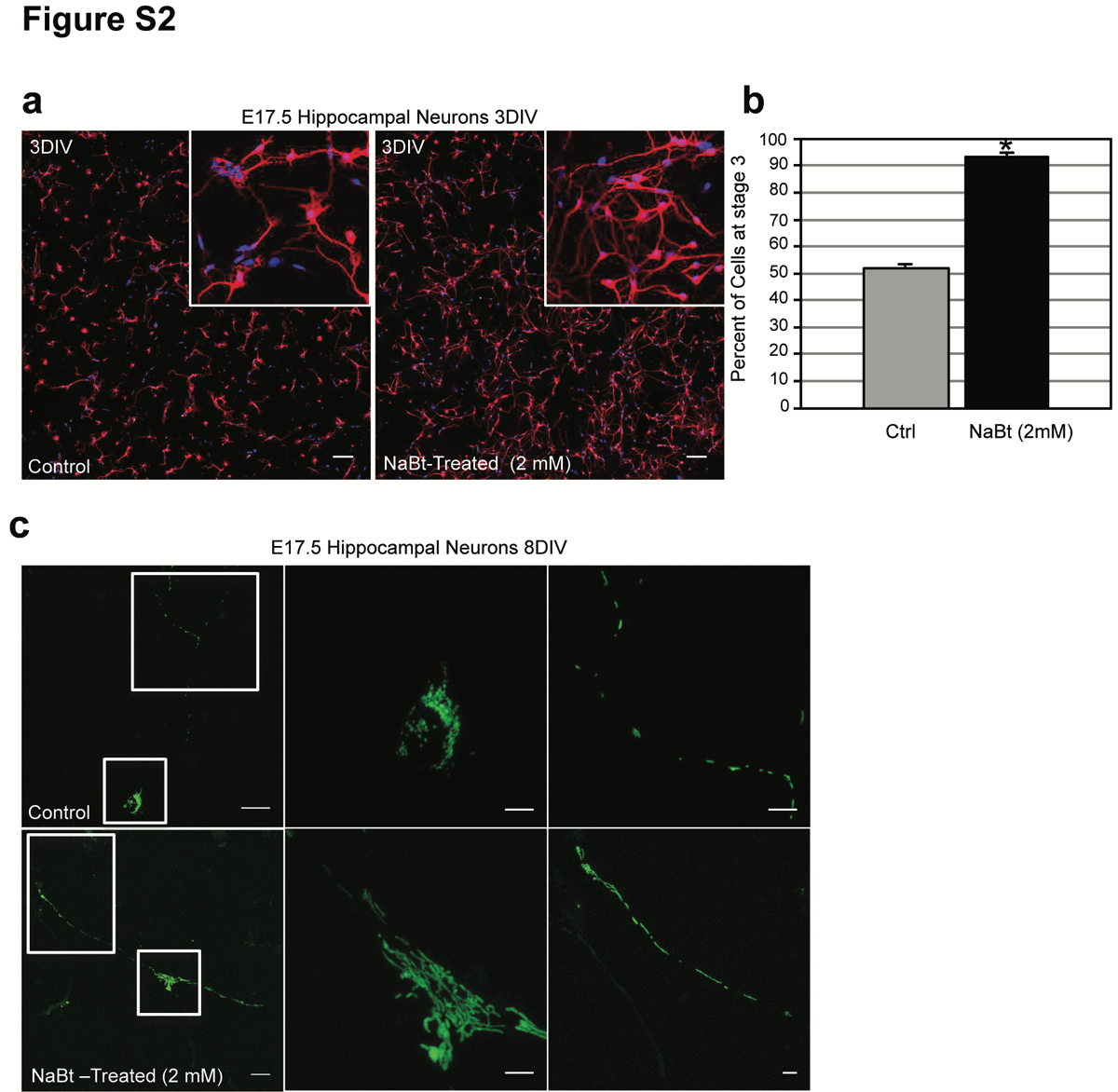


**Supplementary Figure 2:** (**a**) High-resolution tile-stacked confocal micrographs of untreated and NaBt-treated E17.5 hippocampal neurons labeled with β-III tubulin (red) and nuclear counterstain DAPI (blue) at 3DIV with insets showing high magnification of the neuritic network. Scale bar = 20µm. (**b**) Quantification of β-III tubulin^+^ cells having reached the neuronal stage 3. Data are expressed as mean ± S.D. (n= 250 cells for each condition; *p=0.0001). (**c**) Tile-stack confocal micrographs of untreated and NaBt-treated E17.5 hippocampal neurons transfected with the vector mito-GFP at 8div. Scale bar = 10µm. The insets show magnification of boxed soma and processes of untreated and NaBt-treated neurons at 8DIV.


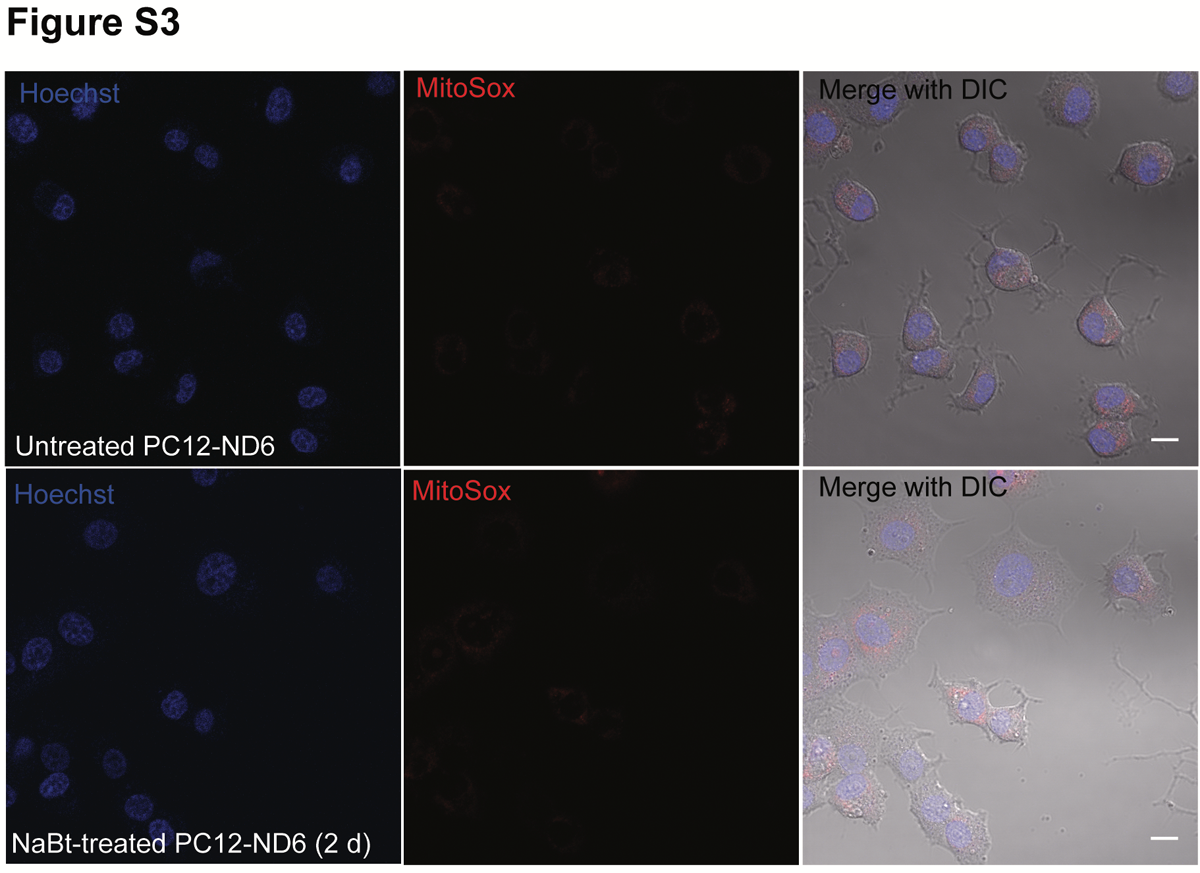


**Supplementary Figure 3:** NaBt does not generate ROS in PC12-ND6 cells. Representative live confocal micrographs of control and NaBt-treated PC12-ND6 cells labeled with the MitoSox™ dye (red) and the nuclear counterstain Hoechst 33342 (blue). Scale bar = 10 µm.


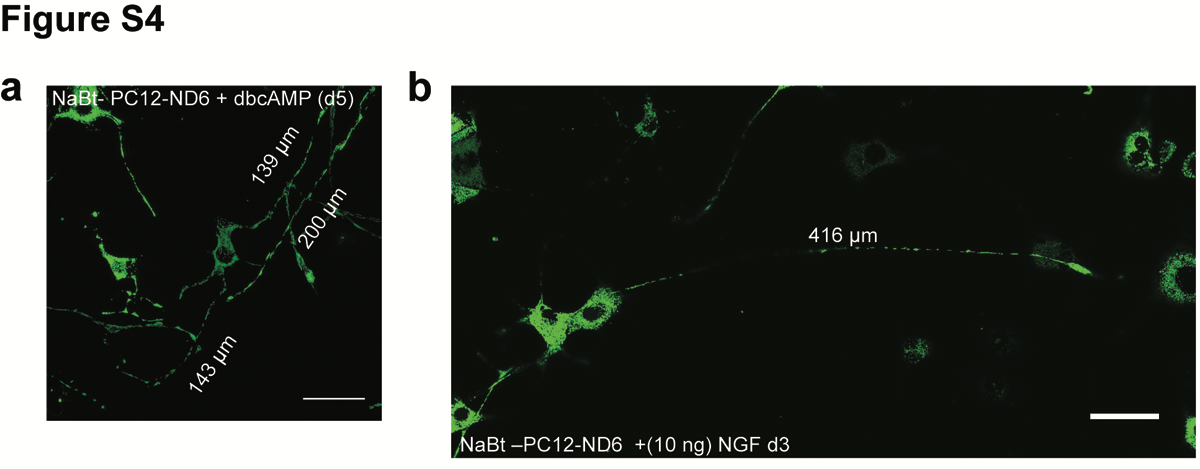


**Supplementary Figure 4:** Pre-treatment of PC12-ND6 cells with NaBt (5 mM) promotes continued neurite growth upon sub-optimal neurotrophic cues. (**a**) PC12-ND6 were first transfected with the mito-GFP vector and treated with NaBt (5 mM) for three days prior to dbcAMP treatment (1 mM) for 5 days. Neurite length is indicated. Scale bar = 50 µm. (**b**) PC12-ND6 cells were transfected and treated with NaBt (5 mM; 3d) and subsequently exposed to sub-optimal concentration of NGF (10 ng/ml) for three days. Neurite length is indicated. Scale bar = 50 µm.


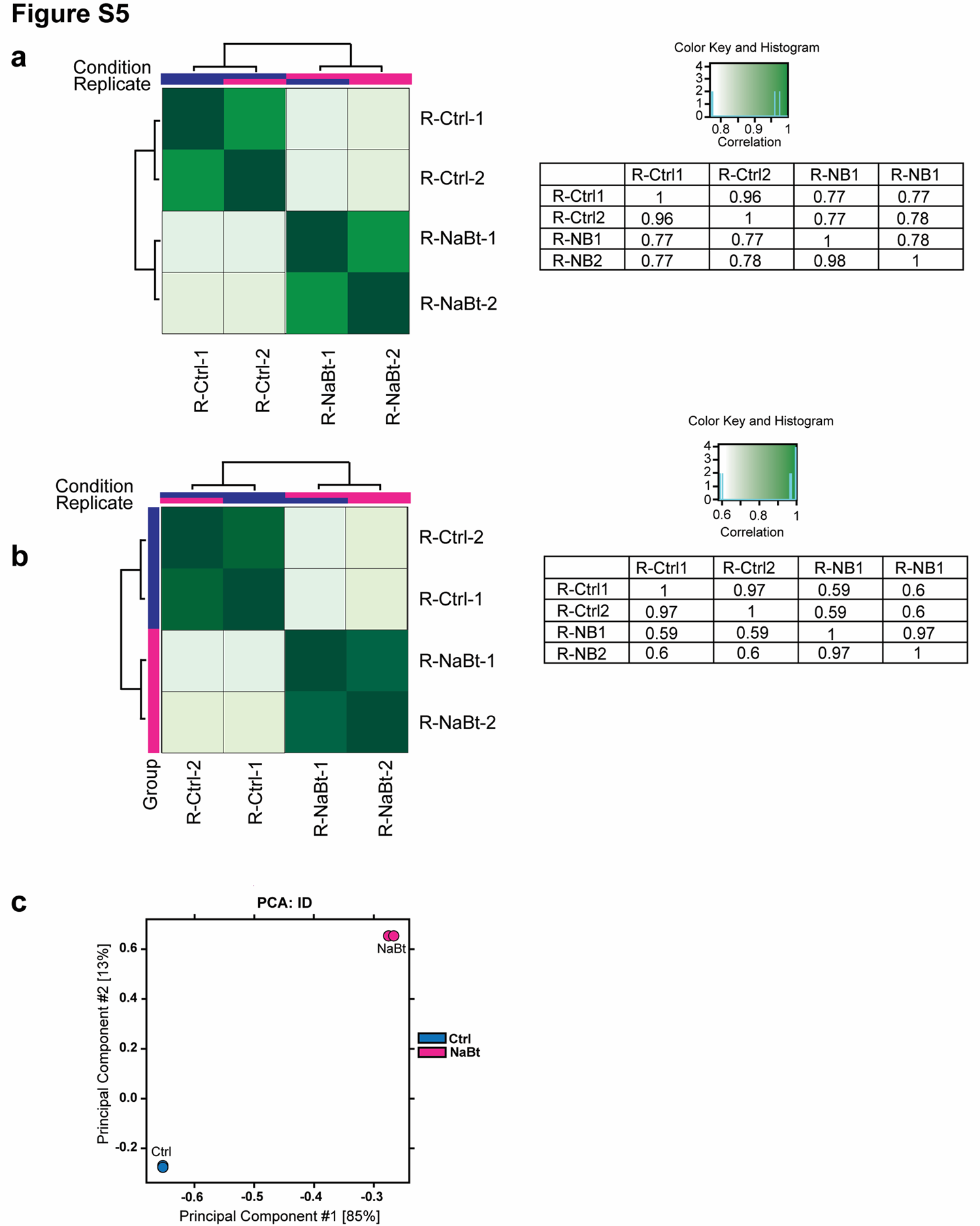


**Supplementary Figure 5:** ChIP-seq controls. (**a**) Correlation heatmap using affinity data (read count) with the corresponding coefficients of correlation indicated in the table. (**b**) Correlation heatmap using only differentially bound sites data with the corresponding coefficients of correlation indicated in the table. (**c**) Principal compment analysis (PCA) of Differentially bound sites in control and NaBt-treated PC12-ND6 cells.
