# Supplementary material for: Epigenetic modifiers promote mitochondrial biogenesis and oxidative metabolism leading to enhanced differentiation of neuroprogenitor cells
Source: Cell Death Dis. 2018 Mar 2;9(3):360. doi: 10.1038/s41419-018-0396-1 (PMC5834638; doi:10.1038/s41419-018-0396-1)
Supplement: Supplementary file 3 — Table S2 [file 41419_2018_396_MOESM3_ESM.docx]

| NCBI Accession Number | Conc_UN | Conc_NB | Fold | p.value | FDR | Name |
| --- | --- | --- | --- | --- | --- | --- |
| NM_080584 | -0.3 | 3.41 | -3.72 | 1.57E-02 | 2.25E-02 | Phkg2 |
| NM_001108879 | -0.3 | 3.66 | -3.97 | 1.08E-02 | 1.58E-02 | Mrpl36 |
| NM_031670 | -0.3 | 3.9 | -4.2 | 3.61E-02 | 4.97E-02 | Napsa |
| NM_020540 | -0.3 | 4.04 | -4.35 | 7.92E-03 | 1.17E-02 | Gstm3 |
| NM_001000796 | -0.3 | 4.22 | -4.53 | 1.39E-03 | 2.20E-03 | Olr1303 |
| NM_001191081 | -0.3 | 4.42 | -4.73 | 4.31E-04 | 7.06E-04 | Cib3 |
| NM_001170484 | -0.3 | 4.5 | -4.81 | 6.08E-04 | 9.84E-04 | Rbm4 |
| NM_172322 | -0.3 | 4.79 | -5.09 | 9.79E-03 | 1.43E-02 | Pycard |
| NM_001100171 | -0.3 | 4.81 | -5.11 | 5.69E-05 | 9.83E-05 | Noxa1 |
| NM_001012224 | -0.3 | 4.95 | -5.25 | 4.94E-05 | 8.57E-05 | Nfe2 |
| NM_053983 | -0.3 | 5.09 | -5.4 | 7.61E-06 | 1.39E-05 | Cd52 |
| NM_001114180 | -0.3 | 5.12 | -5.42 | 9.14E-06 | 1.66E-05 | Plek2 |
| NM_001106175 | -0.3 | 5.43 | -5.73 | 4.88E-07 | 9.48E-07 | Ces2g |
| NM_001107699 | -0.3 | 5.47 | -5.77 | 4.29E-07 | 8.37E-07 | Itga10 |
| NM_080907 | -0.3 | 5.75 | -6.05 | 2.50E-07 | 4.93E-07 | Ppp4r1 |
| NM_021695 | -0.3 | 5.78 | -6.09 | 1.34E-08 | 2.81E-08 | Synpo |
| NM_133386 | 0.69 | 6.88 | -6.19 | 9.47E-16 | 2.61E-15 | Sphk1 |
| NR_133650 | 0.69 | 6.88 | -6.19 | 9.47E-16 | 2.61E-15 | Khps1a |
| NR_133650 | 0.69 | 6.88 | -6.19 | 9.47E-16 | 2.61E-15 | Khps1a |
| NM_001025128 | -0.3 | 5.96 | -6.27 | 2.25E-09 | 4.89E-09 | Gatsl3 |
| NM_001108333 | -0.3 | 5.98 | -6.29 | 1.02E-09 | 2.24E-09 | Rabgef1 |
| NM_001013164 | -0.3 | 6.01 | -6.32 | 5.95E-10 | 1.33E-09 | Kyat1 |
| NM_053503 | -0.3 | 6.11 | -6.41 | 1.60E-10 | 3.65E-10 | Ajuba |
| NM_001015014 | -0.3 | 6.14 | -6.45 | 8.79E-11 | 2.03E-10 | Surf6 |
| NM_001009680 | 0.28 | 6.83 | -6.55 | 1.12E-15 | 3.07E-15 | Oas1i |
| NM_001017451 | -0.3 | 6.25 | -6.56 | 2.02E-10 | 4.58E-10 | Slc35f6 |
| NM_053611 | -0.3 | 6.31 | -6.61 | 1.15E-11 | 2.73E-11 | Nupr1 |
| NM_177426 | -0.3 | 6.44 | -6.74 | 3.21E-12 | 7.81E-12 | Gstm2 |
| NM_001033985 | -0.3 | 6.48 | -6.78 | 3.35E-13 | 8.44E-13 | RT1-CE14 |
| NM_012938 | -0.3 | 6.51 | -6.82 | 2.15E-12 | 5.27E-12 | Ctse |
| NR_128664 | -0.3 | 6.59 | -6.9 | 6.86E-14 | 1.77E-13 | Mirlet7g |
| NM_170667 | -0.3 | 6.72 | -7.02 | 1.31E-14 | 3.47E-14 | Rln3 |
| NM_001034932 | -0.3 | 6.88 | -7.18 | 1.04E-16 | 2.97E-16 | C1qtnf6 |
| NM_001107642 | -0.3 | 6.92 | -7.22 | 5.77E-17 | 1.66E-16 | Tulp1 |
| NM_052805 | -0.3 | 6.95 | -7.26 | 2.43E-17 | 7.10E-17 | Chrna3 |
| NM_001134747 | -0.3 | 7.01 | -7.31 | 6.40E-18 | 1.91E-17 | Zmat4 |
| NM_001191092 | -0.3 | 7.32 | -7.63 | 3.55E-21 | 1.19E-20 | Ccl1 |
| NM_001007720 | -0.3 | 7.34 | -7.64 | 1.62E-21 | 5.49E-21 | Gorasp2 |
| NM_001191894 | -0.3 | 7.43 | -7.74 | 1.02E-20 | 3.36E-20 | Stk32a |
| NM_022632 | -0.3 | 7.48 | -7.79 | 7.24E-23 | 2.55E-22 | Slit2 |
| NM_001106869 | -0.3 | 7.63 | -7.94 | 5.09E-25 | 1.91E-24 | Cdcp1 |
| NM_001000464 | -0.3 | 7.66 | -7.96 | 1.61E-25 | 6.17E-25 | Olr1304 |
| NM_001025421 | -0.3 | 7.66 | -7.96 | 3.84E-25 | 1.45E-24 | Celf1 |
| NM_001000959 | -0.3 | 7.76 | -8.07 | 7.61E-27 | 3.04E-26 | Olr1305 |
| NM_001134592 | -0.3 | 7.94 | -8.25 | 1.30E-28 | 5.49E-28 | Fam160a1 |
| NM_001163164 | -0.3 | 8.08 | -8.39 | 2.83E-31 | 1.29E-30 | Mettl9 |
| NM_001109188 | -0.3 | 8.16 | -8.47 | 2.20E-32 | 1.04E-31 | Lce1f |
| NM_031720 | -0.3 | 8.26 | -8.57 | 7.23E-34 | 3.57E-33 | Dio2 |
| NM_019378 | -0.3 | 8.3 | -8.61 | 2.40E-34 | 1.20E-33 | Srcin1 |
| NM_001107659 | -0.3 | 8.51 | -8.82 | 2.07E-37 | 1.13E-36 | Sema5a |
| NM_053937 | -0.3 | 8.57 | -8.88 | 9.79E-38 | 5.41E-37 | Kcnh6 |
| NM_182669 | -0.3 | 8.68 | -8.99 | 3.17E-40 | 1.90E-39 | Micalcl |
| NM_001105959 | -0.3 | 8.92 | -9.23 | 6.83E-44 | 4.52E-43 | RGD1309104 |
| NM_001107808 | -0.3 | 9.06 | -9.37 | 4.74E-46 | 3.35E-45 | Zbtb46 |
| NM_001134563 | -0.3 | 9.35 | -9.66 | 2.99E-50 | 2.36E-49 | Zfp710 |
| NM_031832 | -0.3 | 10.95 | -11.25 | 4.38E-80 | 7.54E-79 | Lgals3 |
