# Supplementary material for: Epigenetic modifiers promote mitochondrial biogenesis and oxidative metabolism leading to enhanced differentiation of neuroprogenitor cells
Source: Cell Death Dis. 2018 Mar 2;9(3):360. doi: 10.1038/s41419-018-0396-1 (PMC5834638; doi:10.1038/s41419-018-0396-1)
Supplement: Supplementary file 4 — Revised Table S3 [file 41419_2018_396_MOESM4_ESM.docx]

**Supplemental Table 3**

List of antibodies used for immunoblot and immunofluorescence analyses.

| **Antibody** | **Type** | **Source (Cat. No)** | **Dilution** | **Application** |
| --- | --- | --- | --- | --- |
| Acetylated lysine | Rb Ab | Cell Signaling (9441) | 1:200 | ICC |
| β-III tubulin | mAb | Covance (MMS-435-P) | 1:1000 | ICC |
| CBP | Rb Ab | Santa-Cruz (sc-1211 | 1:250 | IB |
| DNA | Mouse mAb | ARP (03-61014) | 1:100 | ICC |
| GAPDH | Mouse mAb | Ambion (AM4300) | 1:20000 | IB |
| MT-COI | Mouse mAb | Mitosciences (MS404) | 1:1000 | IB |
| H2BAc | Rb Ab | Abcam (ab1759) | 1:500 | ICC |
| H3K9Ac | Mouse mAb | Abcam (ab12179) | 1:500 | ICC |
| H3K27Ac | Rb Ab | Abcam (ab4729) | 1:500 | ICC |
| H4K12Ac | Rb Ab | Abcam (ab46983) | 1:500 | ICC |
| NRF-1 | Rb Ab | Abcam (ab34682) | 1:500 | IB |
| SDH-A | Mouse mAb | Mitosciences (MS204) | 1:10000 | IB |
| Tfam | Goat Ab | Santa Cruz (sc-23588) | 1:500 | ICC |
| Tfam | Rb Ab | Biovision (3885-100) | 1:500 | IB |
| Alexa Fluor® 488 anti-rabbit IgG | Goat Ab | Invitrogen (A11034) | 1:1000 | ICC |
| Alexa Fluor® 488 anti-mouse IgG | Goat Ab | Invitrogen (A11029) | 1:1000 | ICC |
| Alexa Fluor® 488 anti-goat IgG | Donkey Ab | Invitrogen (A11055) | 1:1000 | ICC |
| Alexa Fluor® 568 anti-rabbit IgG | Goat Ab | Invitrogen (A11036) | 1:1000 | ICC |
| Alexa Fluor® 568 anti-mouse IgG | Goat Ab | Invitrogen (A11031) | 1:1000 | ICC |
| Alexa Fluor® 647 anti-mouse IgG | Goat Ab | Invitrogen (A21236) | 1:1000 | ICC |
| IRDye® 680LT anti-mouse IgG | Goat Ab | LI-COR (827-11080) | 1:20000 | IB |
| IRDye® 800 anti-rabbit IgG | Goat Ab | LI-COR (827-08365) | 1:20000 | IB |
| IRDye® 680LT anti-mouse IgG1 | Goat Ab | LI-COR (926-68050) | 1:20000 | IB |
| IRDye® 800 anti-mouse IgG2a | Goat Ab | LI-COR (926-32351) | 1:20000 | IB |
