# Supplementary material for: Epigenetic modifiers promote mitochondrial biogenesis and oxidative metabolism leading to enhanced differentiation of neuroprogenitor cells
Source: Cell Death Dis. 2018 Mar 2;9(3):360. doi: 10.1038/s41419-018-0396-1 (PMC5834638; doi:10.1038/s41419-018-0396-1)
Supplement: Supplementary file 5 — Table S4 [file 41419_2018_396_MOESM5_ESM.docx]

**Supplemental Table 4**

List of primers for ChIP-qPCR validation

| Synpo | For: 5’-TCCATCCTCTCCCAGCTGAT-3’  Rev: 5’-GACGTGAACTCATCGGAGCA-3’ |
| --- | --- |
| Ajuba | For: 5’-AGGTTGTCCAGGCTTCCCTA-3’  Rev: 5'-GGGATGTTTGCTAGCTCCCG-3’ |
| Slc35f6 | For: 5’-TCCATCCTCTCCCAGCTGAT-3’  Rev: 5-GACGTGAACTCATCGGAGCA-3’ |
| Gstm2 | For: 5’-GAGCTTGCAGGACTCTGCTC-3’  Rev: 5’-AATGGCGGTTCTTTCTGGGG-3’ |
| Lgals3 | For: 5’-CACTTCAAACAGCACAACCATCC-3’  Rev: 5’-TTAGAACCCAGCCCAAAGCCTA-3’ |
| PosCtrl | For: 5’-CCTGGGAGGCCGTCATTC-3’  Rev: 5’-CCCTGGCTCAGTCCTGTTCT-3’ |
| NegCtrl | For: 5’-GGATAGGGGACAAGGGAGGA-3’  Rev: 5’-AGGGTCCCCTGGTTTCCATA-3’ |
